# Helper T cell immunity in humans with inherited CD4 deficiency

**DOI:** 10.1084/jem.20231044

**Published:** 2024-04-01

**Authors:** Antoine Guérin, Marcela Moncada-Vélez, Katherine Jackson, Masato Ogishi, Jérémie Rosain, Mathieu Mancini, David Langlais, Andrea Nunez, Samantha Webster, Jesse Goyette, Taushif Khan, Nico Marr, Danielle T. Avery, Geetha Rao, Tim Waterboer, Birgitta Michels, Esmeralda Neves, Cátia Iracema Morais, Jonathan London, Stéphanie Mestrallet, Pierre Quartier dit Maire, Bénédicte Neven, Franck Rapaport, Yoann Seeleuthner, Atar Lev, Amos J. Simon, Jorge Montoya, Ortal Barel, Julio Gómez-Rodríguez, Julio C. Orrego, Anne-Sophie L’Honneur, Camille Soudée, Jessica Rojas, Alejandra C. Velez, Irini Sereti, Benjamin Terrier, Nancy Marin, Luis F. García, Laurent Abel, Stéphanie Boisson-Dupuis, Joel Reis, Antonio Marinho, Andrea Lisco, Emilia Faria, Christopher C. Goodnow, Julia Vasconcelos, Vivien Béziat, Cindy S. Ma, Raz Somech, Jean-Laurent Casanova, Jacinta Bustamante, Jose Luis Franco, Stuart G. Tangye

**Affiliations:** 1 Garvan Institute of Medical Research, Darlinghurst, Australia; 2 Faculty of Medicine and Health, School of Clinical Medicine, University of New South Wales Sydney, Sydney, Australia; 3 St. Giles Laboratory of Human Genetics of Infectious Diseases, Rockefeller Branch, The Rockefeller University, New York, NY, USA; 4 Primary Immunodeficiencies Group, Department of Microbiology and Parasitology, School of Medicine, University of Antioquia UdeA, Medellin, Colombia; 5 Laboratory of Human Genetics of Infectious Diseases, Necker Branch, INSERM U1163, Necker Hospital for Sick Children, Paris, France; 6 Paris Cité University, Imagine Institute, Paris, France; 7 Study Center for Primary Immunodeficiencies, Necker Hospital for Sick Children, Assistance Publique–Hôpitaux de Paris, Paris, France; 8 Department of Human Genetics, McGill University, Montreal, Canada; 9 Department of Microbiology and Immunology, McGill University, Montreal, Canada; 10 Dahdaleh Institute of Genomic Medicine, McGill Research Centre on Complex Traits, McGill University, Montreal, Canada; 11 Department of Molecular Medicine, School of Biomedical Sciences, University of New South Wales, Sydney, Australia; 12 Department of Human Immunology, Sidra Medicine, Doha, Qatar; 13 College of Health and Life Sciences, Hamad Bin Khalifa University, Doha, Qatar; 14 The Jackson Laboratory, Farmington, CT, USA; 15 Division of Infections and Cancer Epidemiology, German Cancer Research Center, Heidelberg, Germany; 16 Immunology Department—Pathology, University Hospital Center of Porto, Porto, Portugal; 17 Unit for Multidisciplinary Research in Biomedicine, Institute of Biomedical Sciences Abel Salazar, University of Porto, Porto, Portugal; 18 Service of Internal Medicine, Diaconesse-Croix Saint Simon Hospital, Paris, France; 19 Department of Internal Medicine and Infectious Diseases, Manchester Hospital, Charleville-Mézières, France; 20 Pediatric Immunology-Hematology and Rheumatology Unit, Necker Hospital for Sick Children, Paris, France; 21 Department of Pediatrics and Immunology Service, Edmond and Lily Safra Children's Hospital, Sheba Medical Center, Tel Aviv School of Medicine, Tel Aviv, Israel; 22 San Vicente de Paul University Hospital, Medellin, Colombia; 23 The Genomic Unit, Sheba Cancer Research Center, Sheba Medical Center, Ramat Gan, Israel; 24 National Human Genome Research Institute, National Institutes of Health, Bethesda, MD, USA; 25 Department of Virology, Paris Cité University and Cochin Hospital, Assistance Publique-Hôpitaux de Paris, Paris, France; 26 National Institute of Allergy and Infectious Diseases, National Institutes of Health, Bethesda, MD, USA; 27 Department of Internal Medicine, Cochin Hospital, Assistance Publique–Hôpitaux de Paris, Paris Cité University, Paris, France; 28 Cellular Immunology and Immunogenetics Group, University of Antioquia UdeA, Medellin, Colombia; 29 Dermatology Service, University Hospital Center of Porto, Porto, Portugal; 30 School of Medicine and Biomedical Sciences, University of Porto, Porto, Portugal; 31 Department of Clinical Immunology, University Hospital Center of Porto, Porto, Portugal; 32 Allergy and Clinical Immunology Department, University Hospital Center of Coimbra, Coimbra, Portugal; 33 Howard Hughes Medical Institute, New York, NY, USA; 34 Department of Pediatrics, Necker Hospital for Sick Children, Assistance Publique–Hôpitaux de Paris, Paris, France

## Abstract

CD4^+^ T cells are vital for host defense and immune regulation. However, the fundamental role of CD4 itself remains enigmatic. We report seven patients aged 5–61 years from five families of four ancestries with autosomal recessive CD4 deficiency and a range of infections, including recalcitrant warts and Whipple’s disease. All patients are homozygous for rare deleterious *CD4* variants impacting expression of the canonical CD4 isoform. A shorter expressed isoform that interacts with LCK, but not HLA class II, is affected by only one variant. All patients lack CD4^+^ T cells and have increased numbers of TCRαβ^+^CD4^−^CD8^−^ T cells, which phenotypically and transcriptionally resemble conventional Th cells. Finally, patient CD4^−^CD8^−^ αβ T cells exhibit intact responses to HLA class II–restricted antigens and promote B cell differentiation in vitro. Thus, compensatory development of Th cells enables patients with inherited CD4 deficiency to acquire effective cellular and humoral immunity against an unexpectedly large range of pathogens. Nevertheless, CD4 is indispensable for protective immunity against at least human papillomaviruses and *Trophyrema whipplei*.

## Teaser

This study describes clinical features and cellular and molecular mechanisms underlying immune deficiency in seven patients with biallelic germline variants in *CD4*. The data reveal important roles for CD4 in host defense against a range of pathogens, particularly human papilloma virus.

## Introduction

The role of human CD4^+^ αβT cells in host defense has been delineated by the natural history of patients with two immunodeficiencies that affect the numbers of these cells in peripheral blood: acquired immune deficiency syndrome (AIDS), secondary to human immunodeficiency virus (HIV) infection (Dalgleish et al., 1984; Klatzmann et al., 1984; Maddon et al., 1986), and inherited MHC class II deficiency due to inborn errors of transcription factors that govern expression of these human histocompatibility leukocyte antigens (HLA) loci (Nekrep et al., 2003). HIV/AIDS was first described in 1981 (Centers for Disease Control, 1981; Gottlieb et al., 1981), and CD4 was soon identified as the main receptor for viral entry into CD4^+^ T cells (Dalgleish et al., 1984; Klatzmann et al., 1984; Maddon et al., 1986), the predominant host cells for HIV (Wilen et al., 2012). Monitoring CD4^+^ T cell numbers in HIV-infected patients became a key predictor of disease progression and severity. This was underscored by the discovery of rare HIV-infected patients who did not progress to AIDS and maintained significant numbers of CD4^+^ T cells for many years after infection (Buchbinder et al., 1994; Cao et al., 1995; Lambotte et al., 2005; Rinaldo et al., 1995). The loss of CD4^+^ T cells in HIV infection correlated with susceptibility to a large number of infectious agents including viruses, bacteria, fungi, and parasites, which are life-threatening in untreated patients (Gottlieb et al., 1981; Masur et al., 1981; Small et al., 1983; Vieira et al., 1983). Nevertheless, HIV-infected individuals have many immunological abnormalities in addition to CD4^+^ T cell lymphopenia (Moir et al., 2011), preventing the generalization of direct mechanistic links of CD4^+^ T cell deficiency with specific infectious diseases.

HLA class II deficiency is a rare inborn error of immunity (IEI) first described in 1983 (Lisowska-Grospierre et al., 1983). It is caused by bi-allelic deleterious variants in *CIITA*, *RFANXK*, *RFX5*, or *RFXAP*, which all encode transcription factors necessary for constitutive and inducible expression of HLA class II on hematopoietic and non-hematopoietic antigen-presenting cells (APCs) (Nekrep et al., 2003). Cognate interactions between CD4 on T cells with HLA class II on thymic epithelium and peripheral APCs are critical for thymic selection and the peripheral function of CD4^+^ T cells, respectively (Ouederni et al., 2011). Thus, due to disrupted CD4/HLA class II interactions in the thymus, individuals with HLA class II deficiency have profound CD4^+^ T cell deficiency (10–20-fold reduction) and are consequently susceptible to a broad range of pathogens (Al-Herz et al., 2013; Ben-Mustapha et al., 2013; Ouederni et al., 2011; Rozmus et al., 2013). The only curative treatment for HLA class II deficiency is allogeneic hematopoietic stem cell transplantation, suggesting that hematopoietic APCs in the thymus can be sufficient for negative and positive selection during thymopoiesis (Aluri et al., 2018; Lum et al., 2019). Inherited HLA class II deficiency due to mutations in *CIITA*, *RFANXK*, *RFX5*, or *RFXAP* has therefore revealed essential roles for CD4^+^ T cells in host defense against a myriad of infectious agents. HLA class II deficiency is complete in most patients. However, the CD4^+^ T cell defect is not absolute inasmuch that CD4^+^ T cell development and T-dependent humoral immunity are inexplicably impaired, but not abolished, in affected individuals (Mach et al., 1996; Saleem et al., 2000). Moreover, the unique and fundamental role(s) of the CD4 co-receptor itself in human T cell selection and development (Glassman et al., 2018), as well as in host defense against infection and immune regulation, remains incompletely resolved.

Recently, two patients with biallelic germline deleterious variants in *CD4* have been reported (Fernandes et al., 2019; Lisco et al., 2021). The first study identified an intronic essential splice site substitution homozygous variant in a 45-year-old Portuguese woman born to consanguineous parents who developed recalcitrant human papillomavirus (HPV)–related warts on her feet and hands (Fernandes et al., 2019). The second study reported a homozygous start loss *CD4* variant in a 22-year-old white American woman born to consanguineous parents who presented with pneumonia and a history of recalcitrant HPV-related warts on her trunk and extremities (Lisco et al., 2021). Flow cytometry revealed that both patients lacked T cells expressing CD4 (Fernandes et al., 2019; Lisco et al., 2021). Despite this, when compared with patients with HIV/AIDS or HLA class II deficiency, infections in these two adults were surprisingly narrow and relatively mild. In both cases however, the patients’ lymphocytes were characterized by an expanded population of CD4^−^CD8^−^TCRαβ^+^ T cells, which were proposed to retain some functional characteristics of typical CD4^+^ T cells (Lisco et al., 2021). The nature of these cells and the mechanisms underlying susceptibility and resistance to infectious agents remain unknown. Moreover, the molecular and cellular consequences of the rare *CD4* variants reported have not been assessed. We have now studied seven patients from five unrelated kindreds of four ancestries, including the two previously published patients (Fernandes et al., 2019; Lisco et al., 2021), with biallelic rare variants in *CD4* and a lack of detectable CD4-expressing peripheral blood T cells.

## Results

### Homozygosity for rare *CD4* variants

**Figure 1.**
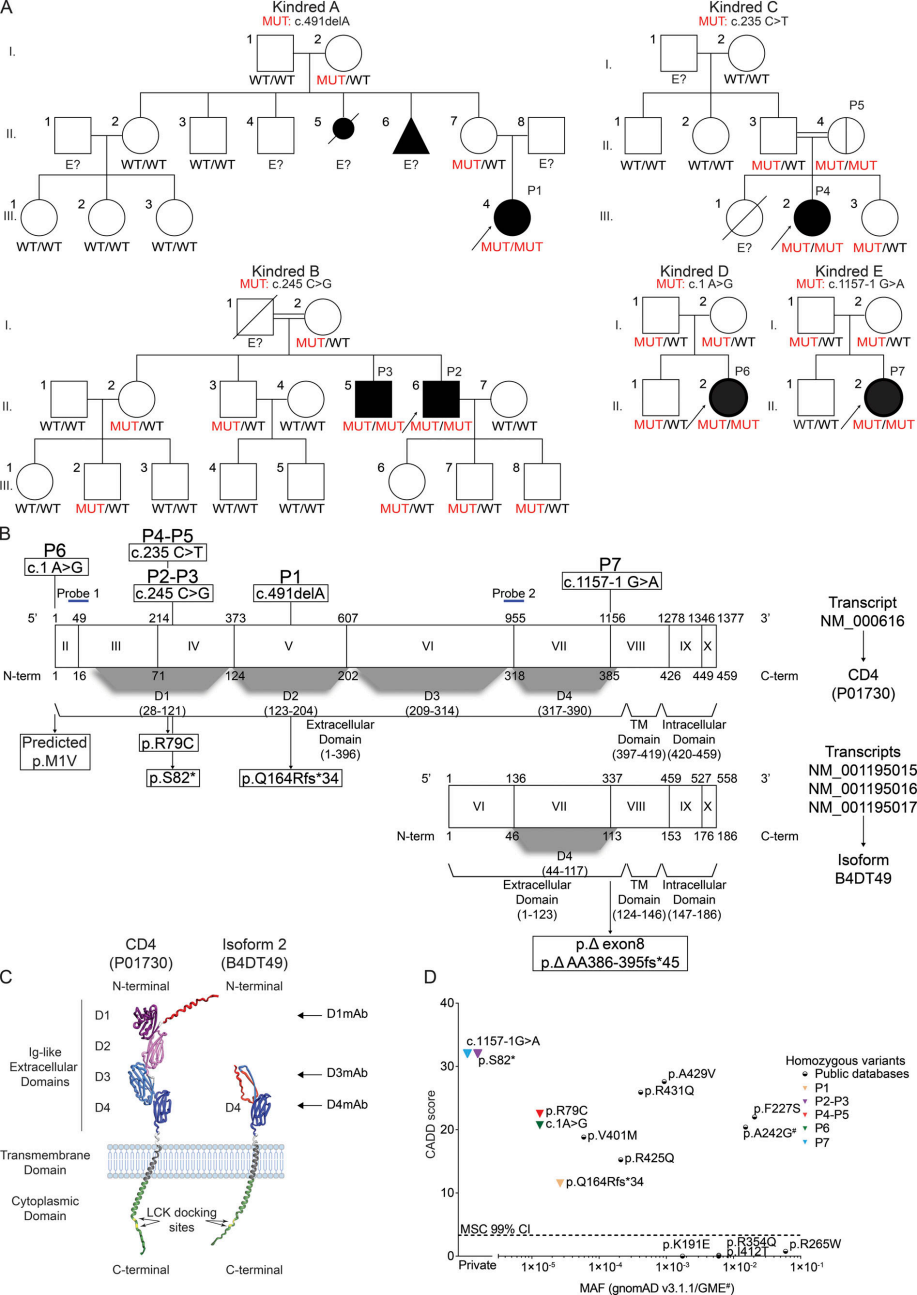
**Autosomal recessive CD4 deficiency. (A)** Pedigree showing familial segregation of c.491delA (Kindred A), c.245C>G (Kindred B), c.235C>T (Kindred C), c.1A>G (Kindred D), and c.1157-1G>A (Kindred E). Individuals of unknown genotype are labeled "E?". **(B)** Schematic representation of four *CD4* transcripts and their two corresponding isoforms. Exon numeration is based on NM_000616. Nucleotide (above) and amino acid (below) numeration are indicated based on each transcript/isoform. Protein domains are represented below each isoform. Patients’ variants are represented on NM_000616/CD4 (P01730). Two qPCR probes used in this study are represented by blue lines (probe 1: junction exon II–III; probe 2: junction exon VI–VII). **(C)** Alphafold representation CD4 and isoform 2 expressed at the cell surface with signal peptide (red), Ig-like extracellular domains (D1: purple; D2: pink; D3: light blue; D4: dark blue), transmembrane domain (dark gray), and cytoplasmic domain (green) with LCK docking sites (yellow). The different mAbs used in the study are also represented. **(D)** Minor allele frequency (MAF) and combined annotation-dependent depletion (CADD) score for all *CD4* variants reported homozygous in public databases (circles) and found in patients (triangles). The mutation significance cutoff (MSC, 99% confidence interval [CI]) is represented by the dotted line. # indicates a variant only found in the GME database.

**Figure S1.**
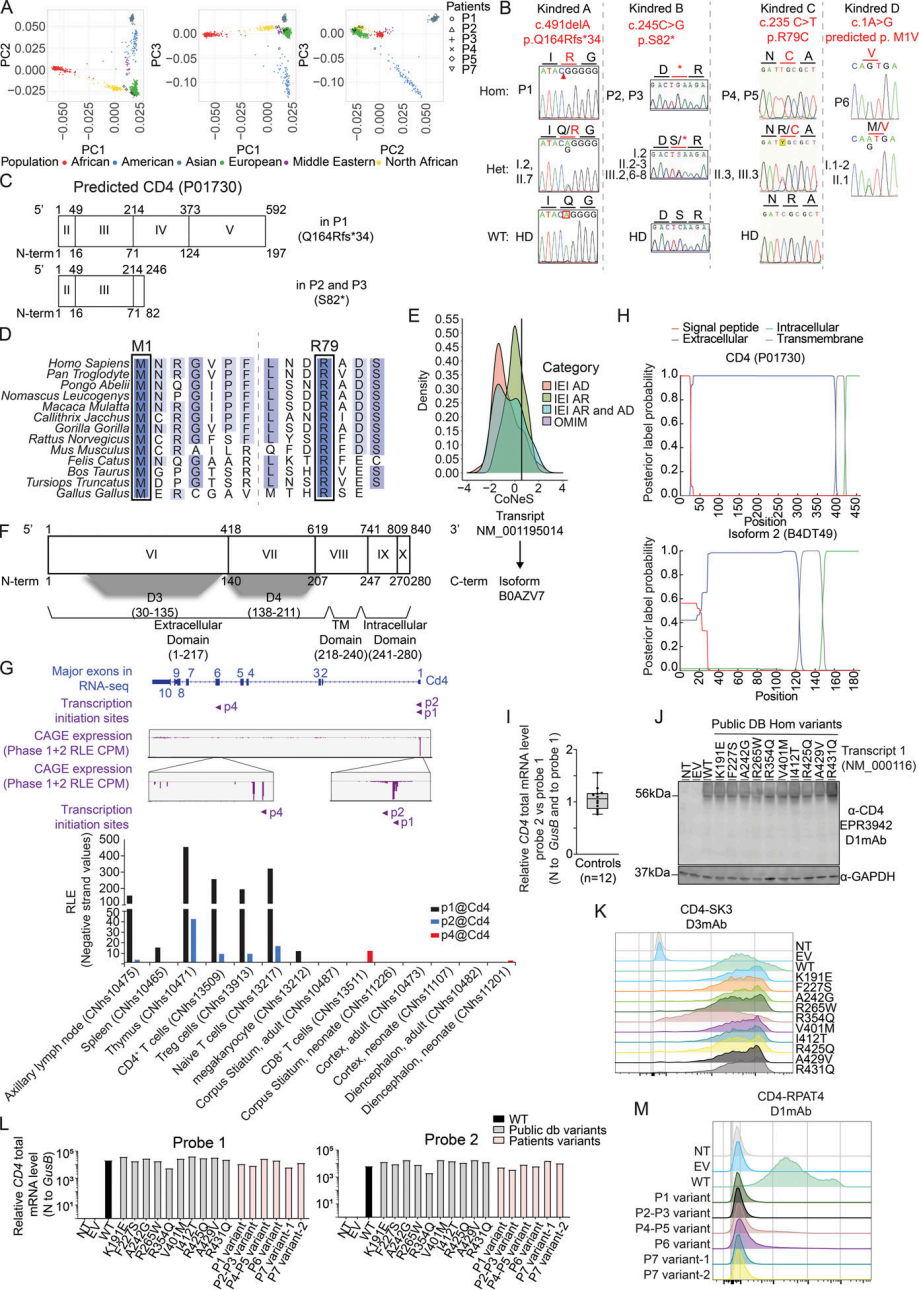
**Genetics, in silico analysis, and impact of identified *CD4* variants on mRNA and protein expression. (A)** Principal component analysis of WES data from the patients and our in-house WES database. **(B)** Electropherograms of representative *CD4* nucleotide sequences in Kindreds A–D. **(C)** Schematic representation of the predicted truncated CD4 in P1–P3. **(D)** Alignment of CD4 M1 and R79 residues in humans and 11 other representative animal species. Dark blue: highly conserved; blue: well conserved; light blue: moderately conserved; white: not conserved. **(E)** CoNeS of *CD4*. AD: autosomal dominant, AR: autosomal recessive. **(F)** Schematic representation of *CD4* NM_001195014 and its corresponding isoform (B0AZV7). Exon numeration is based on NM_000616. Nucleotide (above) and amino acid (below) numeration is indicated. Protein domains are represented below isoform. **(G)** *Cd4* transcript expression in mouse tissues. Top: CAGE-seq relative expression track from the FANTOM5 project showing signal pool for all tested tissues. Three transcriptional initiation sites are detected. p1 and p2 are located upstream of exon 1, while p4 is upstream of exon 6. Bottom: Bar graph showing CAGE-seq relative expression for representative tissues. Note that p4 is only detected in the brain. **(H)** Prediction of transmembrane topologies and signal peptides done by Phobius (https://phobius.sbc.su.se/) based on CD4 and isoform 2 amino acid sequences. Red: signal peptide; blue: extracellular domain; green: Intracellular domain; gray: transmembrane domain. Y axis represents probability, and x axis represents amino acid prediction. **(I)** *CD4* total mRNA level in healthy donors relative to *GUS* (dCT). For each sample, ddCT was calculated as follows: probe 2 dCT normalized to probe 1 dCT value. **(J)** Immunoblotting with N-terminal CD4 D1mAb (EPR3942) and GAPDH on total cell lysate from HEK293T either non-transfected (NT) or transiently transfected with an empty vector (EV) or with vectors encoding the indicated *CD4* transcript. **(K)** Flow cytometry following extracellular staining with CD4 (D3mAb; SK3) of HEK293T either non-transfected (NT) or transiently transfected with an empty vector (EV) or with vectors encoding the indicated *CD4* transcript. **(L)** Relative *CD4* total mRNA level (probe 1 and 2) normalized to *GUS* of HEK293T either non-transfected (NT) or transiently transfected with an empty vector (EV) or with vectors encoding the indicated CD4 transcript. **(M)** Flow cytometry following extracellular staining with CD4 (D1mAb; RPAT4) of HEK293T either non transfected (NT) or transiently transfected with an empty vector (EV) or with vectors encoding the indicated *CD4* transcript. Data are representative of at least two independent experiments. Source data are available for this figure: SourceData FS1.

We investigated seven HIV-negative patients (P1–P7) who lacked detectable CD4^+^ T cells in their peripheral blood. P6 and P7 were published previously by Lisco et al. (2021) and Fernandes et al. (2019), respectively. The patients are from five unrelated families of Colombian (Kindred A), Portuguese (Kindred B and E), Palestinian (Kindred C), and presumed white American (Kindred D) descent (Fig. 1 A and Table 1). Whole-exome sequencing (WES) was performed on all newly reported patients (P1–5) and P7, and an autosomal recessive (AR) model of inheritance was tested. Note that the P6 variant has been identified with a candidate gene sequencing approach. High homozygosity rates for P1 (4.3%), P2 (3.7%), P3 (3.4%), P4 (11.6%), P5 (3%), and P7 (5%) confirmed they were all born to unrelated but each consanguineous parents (Belkadi et al., 2016). Principal component analysis (PCA) confirmed P1 to be of Latin American origin, P2, P3, and P7 of European descent, while the origin of P4 and P5 lies between European and North African (Fig. S1 A). For P1–P5, homozygous non-synonymous rare coding variants predicted to be damaging, and in known IEI genes associated with reduced numbers of CD4^+^ T cells were selected. Homozygous variants in *CD4* were identified in each patient and confirmed by Sanger sequencing (Fig. 1 A and Fig. S1 B). P1 carried a homozygous frameshift deletion (c.491delA) in exon 5, predicted to introduce a premature stop codon (p.Q164Rfs*34). The father of P1 was unavailable for genetic testing. However, P1’s mother (II.6) and maternal grandmother (I.2) were found to be heterozygous carriers of this variant (Fig. 1 A and Fig. S1 B). P2 and P3 were homozygous for a nonsense variant (c.245C>G) in exon 4 that introduced a premature stop codon at amino acid position 82, replacing a serine residue (p.S82*). We also detected a homozygous missense variant (c.493G>T) in exon 5 of *CD4* in P2 and P3 which replaces glycine at position 165 with a tryptophan (p.G165W). As this missense variant is downstream of the p.S82* nonsense variant, it was not studied further. Seven relatives of P2 and P3 (I.2, II.2, II.3, III.2, III.6, III.7, and III.8) were found to be heterozygous carriers of the nonsense variant (Fig. 1 A and Fig. S1 B) whereas four others were wild-type (WT; III.1, III.3, III.4, III.5). P4 and P5 carry a homozygous missense variant (c.235C>T) in exon 4, replacing arginine at position 79 with cysteine (p.R79C) (Fig. 1, A and B; and Fig. S1 B). Two other relatives of P4 were heterozygous carriers of this variant (II.3 and III.3). P6 is homozygous for a missense variant in the translation-initiation codon (c.1A>G, predicted p.M1V) (Fig. 1, A and B; and Fig. S1 B) (Lisco et al., 2021). P7 is homozygous for an intronic substitution affecting the last base pair (bp) of intron 7 (c.1157-1G>A) (Fig. 1, A and B). The disruption of the acceptor splice site results in two frameshift deletions: c.1157_1278del (122 bp; exon 8 skipping) and c. 1157_1185del (29 bp; use of alternative acceptor site) resulting in premature stop codons at position p.V386_R426delfs*13 and p.V386_Q395delfs*45, respectively (Fernandes et al., 2019). Notably, none of the patients carried the *CD4* polymorphism (c.868 C>T; p.R240W) that disrupts detection of CD4 by the well-characterized OKT4 (T4) anti-CD4 mAb (data not shown) (OMIM +186940) (Hodge et al., 1991; Lederman et al., 1991; Takenaka et al., 1993).

**Table 1.** Genetic, demographic, weight and height, and clinical spectrum of patients with AR CD4 deficiency

									Weight and height			
Patient/Kindred	Mutation	Origin	Gender (F/M)	Year of birth	Follow-up	Age at onset of symptoms	Clinical infectious phenotype (infections, microbiology, pathology results) Current treatment prophylaxis	Detection of auto-antibodies/age	Age (yr)	Weight (kg) [Z score]	Height (cm) [Z score]	BMI (kg/m^2^)
P1/A	c.491delA/491delA p.Q164fs197*/Q164fs197*	Colombia	F	2003	Alive	2 mo	BCG-vaccine: No AE Other alive vaccine: MMR, YFV without AE Failure to thrive, diarrhea Multiple pneumonia, chronic sinusitis, otitis Endophtalmitis, iridocyclitis Urinary tract infection Varicella without complications 3C syndrome^b^ Prophylaxis: Ig and ATB	N.D.	0 3 5 6.5 7 8.6 9.2 13 15 16	2.44 (−1) 11 (−1) 14 (−2) 17 (−1) 18 (−1) 15 (>−2) 18 (−2) 38 (−2) 38.5 42	48 (−2) 86 (>−2) 95 (>−2) 102 (>−2) 104 (>−2) 109 (>−2) 112 (>−2) 116 (>−2) 141 141	ND ND ND ND ND ND ND ND 19.5 (normal) 21.1 (normal)
P2/B P3/B	c.245C>G/245C>G p.S82*/S82* c.245C>G/245C>G p.S82*/S82*	Portugal^a^ Portugal	M M	1962 1966	Alive Alive	49 yr 10 yr	BCG vaccine: No AE Other alive vaccine: None At 49 yo: Colon polyps with dysplasia At 52 yo: Diagnosis of classic Whipple’s disease (diarrhea, abdominal pain, arthritis). *T. whipplei* (+) in culture and PCR saliva, stool, and synovial fluid, PAS(+). Treatment: Hydroxychloroquine and doxycycline Oral labial herpes Warts Prophylaxis: None BCG vaccine: No AE Other alive vaccine: None At 10 yo: Pulmonary tuberculosis At 20 yo: Laryngeal tuberculosis At 46 yo: Dental abscess requiring surgical drainage Hiatal hernia and erythematous gastritis Since adolescence: Multiple non-pruritic verrucous skin lesions (by HPV3+) and genital warts (by HPV31). Treatment: Isotretinoin with good response Prophylaxis: None	At 52 yo: ANA (−) ANCA (−) Anti-dsDNA (−) Anti SS-B (−) Anti-J01 (−) Anti-RNP (−) Anti-Scl70 (−) Anti-smith (−) Anti-SSA (−) At 50 yo: ANA (−) ANCA (−) Anti-dsDNA (−) Anti SS-B (−) Anti-RNP (−) Anti-smith (−) Anti-SSA (−)	61 57	90 70	163 175	33.9 (obesity) 22.9 (normal)
P4/C P5/C	c.235C>T/235C>T p.R79C/R79C c.235C>T/235C>T p.R79C/R79C	Palestinian Palestinian	F F	2018 2000	Alive Alive	2 mo N.A.	BCG-vaccine: BCG-itis at 3 mo Failure to thrive Recurrent pulmonary infections without microbe isolation, requiring hospitalizations Cryptosporidiosis infection and oral candidiasis Prophylaxis: Fluconazole and TMP/SMX	N.D.	0 1 6 23	3.2 (0) 10 (0) 17 (−1) 51	ND ND ND ND	ND ND ND ND
P6/D	c.1A>G/1A>G Predicted p.M1V/M1V	White American	F	1997	Alive		BCG vaccine: No AE Other alive vaccine: MMR, polio IPV/OPV, varicella zoster (Varivax) without AE Respiratory syncytial virus pneumonia requiring inpatient admission (1 mo old) -At 1 yo: Rotavirus gastroenteritis -At 5–6 yo: Diagnosis of attention deficit hyperactivity disorder and currently managed with lisdexamfetamine -Until 4–5 yo: Recurrent otitis media requiring myringotomy tubes (nine procedures) in childhood (six episodes per yr until age 4–5) -Recurrent episodes of upper respiratory infection with productive cough (4–5 episodes per yr): empiric course of antimicrobials, tonsillectomy, and adenoidectomy. -Multiple episodes of transient cervical lymphadenopathy during childhood. -At 5–6 yo: Onset of multiple skin warts recalcitrant to topical treatment, cryotherapy, and surgical excision. Spontaneously resolved around age 18–19. -At 6 yo: Varicella despite receiving first dose of varicella vaccine at age 1 -At 15 yo: Recurrent episodes of back pain with imaging significant for intervertebral herniations in the lower thoracic spine -At 22 yo: Acute presentation with multifocal pneumonia and hypoxic respiratory failure requiring mechanical ventilatory support. A nasopharyngeal molecular assay tested positive for rhino-virus and enterovirus.	Anti β2-glycoprotein 1 AB panel (IgG and IgM) (−) Anti-thyroglobulin (−) Thyroid peroxidase AB (−) Anti-proteinase 3 (−) Anti-MPO (−) Anti-dsDNA (−) ANA (−) anti-ENA (−) ACA IgG and IgM (−) Rheumatoid factor (−) Anti-CCP (−)	22 25	107.4 111	168.8 169	37.6 (obesity) 38.9 (obesity)
P7/E	c.1157-1G>A/1157-1G>A p.V386_R426delfs*13 and p.V386_Q395delfs*45	Portugal	F	1974	Alive	10 yr	Exuberant and disfiguring warts in both feet and hands. Warts were refractory to treatment with keratolytic agents, cryosurgery, and excision, with minor improvement after treatment with acitretin in association with topical 50% urea cream. Past medical history of measles and mumps during her infancy and varicella infection during her first pregnancy, which all resolved without complications Allergic rhino-conjunctivitis treated with cetirizine and fluticasone, and chronic polyarthralgias in the absence of impaired functionality HIV 1/2 (−), HTLV-1 (−), syphilis (−)	Anti-neutrophil (−) Anti-ds-DNA (−)	49	64	150	28.44 (overweight)

Mutations, patient numbers, and family numbers are as in Fig. 1. Consanguinity, patient origin, sex, current vital status, age at onset of symptoms, and clinical phenotype are shown. AE: adverse effect, ATB: antibiotics, BB-B: complement factor B, ANCA: anti-neutrophils cytoplasmic autoantibody, dsDNA: double-stranded (ds, native) DNA, ANA: antinuclear antibodies, PAS: periodic acid-Schiff, MMR: measles-mumps-rubella, YFV: yellow fever vaccine, ND: not determined; BMI: body mass index, TMP: trimethoprim, SMX: sulfamethoxazole.

a P2 lives in France.

b Clinical manifestations are mild cardiac abnormalities, interatrial (IAC) and interventricular communication (IVC), absent cerebellar vermis, congenital glaucoma with megalocornea, facial dysmorphism, and mild mental retardation.

### *CD4* population genetics

The homozygous *CD4* variants identified in patients P1–P6 are localized to exons encoding the distal extracellular domains of CD4 (D1 and D2 domain, NM_000116), while the variant in P7 affects the transmembrane (TM) region of CD4 (Fig. 1, B and C). S82* (P2, P3), Q164Rfs*34 (P1) (Fig. S1 C), and V386_R426delfs*13 (P7) (Fernandes et al., 2019) are predicted to encode truncated proteins that, if expressed, would lack the TM domain, thus being produced as soluble truncated proteins. The M1 (P6) and R79 (P4, P5) residues are highly conserved in CD4 from 12/12 representative species (Fig. S1 D). Each of the variants has a combined annotation-dependent depletion (CADD) score well above the mutation significance cutoff (MSC) for *CD4* (3.313) (Fig. 1 D) (Itan et al., 2016; Kircher et al., 2014). Furthermore, *CD4* has a gene damage index of 2.96, a neutrality index score of 0.50, as well as a consensus negative selection (CoNeS) score of 0.57 (Fig. S1 E), and a supervised CoNeS (SCoNeS) of 0.942 (Itan et al., 2015; Rapaport et al., 2021). These characteristics indicate that *CD4* has evolved under modest purifying selection and CD4 deficiency is more likely AR than autosomal dominant (Rapaport et al., 2021). The S82* (P2, P3) and c.1157-1G>A (P7) variants are private, being absent from public databases (gnomAD [Karczewski et al., 2020], GME Variome [Scott et al., 2016], TOPMed Bravo [Taliun et al., 2021]). c.1A>G (P6), R79C (P4, P5), Q164Rfs*34 (P1), and G165W variants were reported in gnomAD (v3.1.1) but only in a heterozygous state and with minor allele frequencies (MAF) between 1.31 × 10^−5^ and 2.63 × 10^−5^ (Table S1). 10 additional homozygous *CD4* missense variants were present in gnomAD (K191E, F227S, R265W, I412T, A429V, R431Q), ATAVDB (R354Q), TOPMed Bravo (V401M), or GME Variome (A242G, R425Q) databases with MAFs ranging from 5.91 × 10^−5^ to 5.96 × 10^−2^ (Fig. 1 C). There were no homozygotes for any predicted loss-of-function variant in any public database. Altogether, population genetic analyses revealed the *CD4* variants identified in all patients (P1–P7) to be strong candidates for detailed biochemical and functional investigation to establish causality.

### Clinical features of the patients homozygous for rare *CD4* variants

Detailed clinical features of all patients, including infectious history and growth/weight over time, are provided in Table 1. P1 (Kindred A), a Colombian girl born in 2003, was diagnosed at 2 mo of age with Ritscher-Schinzel or 3C syndrome (OMIM #220210) (due to a homozygous variant in a novel 3C-causing gene, to be reported elsewhere) (Elliott et al., 2013; Leonardi et al., 2001; Ritscher et al., 1987; Voineagu et al., 2012). She presented with mild cardiac (interatrial and interventricular communication) and neurological abnormalities (absent cerebellar vermis), congenital glaucoma with megalocornea, facial dysmorphism, and mild mental retardation (Pira-Paredes et al., 2017). She exhibited failure to thrive and developed recurrent infectious episodes in her early years including multiple pneumonia, chronic sinusitis, urinary tract infection, diarrhea, iridocyclitis, and endophthalmitis. At the last follow-up, in 2020, aged 17 years, P1 was alive and well.

P2 and P3 (Kindred B) are Portuguese brothers born in 1962 and 1966, respectively. In 2017, at age 52 years old (yo), the proband (P2) presented with recurrent fever, diarrhea, asthenia, hiatus hernia with vascular ectasia, oral labial herpes, warts, enteral ulceration, and joint pain affecting his knees and wrists. P2 was diagnosed with Whipple disease following the detection of *Tropheryma whipplei* in his duodenum, stool, and saliva by PCR. Treatment with doxycycline and hydroxychloroquine was effective. In 2022, his PCR for *T. whipplei* in blood, saliva, and stool was negative. P3 developed pulmonary tuberculosis at 10 yo and laryngeal tuberculosis at 20 yo. Since 9 yo, he also presented with multiple non-pruritic verrucous skin lesions (HPV3^+^) and genital warts (HPV31^+^). In 2019, an evaluation for Whipple disease was made but PCR for *T. whipplei* in blood, saliva, and stool was negative.

P4 (Kindred C), a Palestinian girl born in 2013, was hospitalized three times during her first year of life due to a recurrent chest infection of unknown etiology and failure to thrive. She also had a prolonged local reaction to the Bacillus Calmette-Guérin (BCG) vaccine. At 11 mo, she presented with chronic diarrhea and was also diagnosed with cryptosporidiosis infection and oral candidiasis. The mother, P5 (Kindred C, Table 1), a Palestinian woman born in 2000, is healthy without severe infections and is not on any medications.

P6 (Kindred D) is a 22-yo white American woman with a history of recalcitrant HPV-related warts on her trunk and extremities, recurrent otitis, and episodes of upper respiratory infections (Lisco et al., 2021). At 17 yo, she developed severe multifocal pneumonia due to rhinovirus and enterovirus requiring mechanical respiratory assistance.

P7 (Kindred E) is a 49-yo Portuguese woman with a history of persistent, extensive, and refractory warts in both feet and hands since the age of 10 years (Fernandes et al., 2019).

### In silico studies of *CD4* transcripts

In public databases, the human *CD4* gene is predicted to encode three isoforms (Fig. 1 B and Fig. S1 F) generated from five different transcripts (hg19; reviewed UCSC genes tracks; http://www.genome.ucsc.edu/). One transcript (NCBI Refseq variant 1: NM_000616) is comprised of 10 transcribed exons (initiation codon in exon 2) and encodes a 458–amino acid (AA) protein representing CD4 per se (UniProt: P01730) (Fig. 1 B). The other four transcripts use an alternative splice site and lack stretches corresponding to two consecutive exons (exon 2 and 3), resulting in translation being initiated from downstream alternative in-frame start sites located in exon 6 (Fig. 1 B and Fig. S1 F). Interestingly, the highest degree of homology between the murine *Cd4* gene (10 exons and 9 introns) (Rahemtulla et al., 1991) and human *CD4* is found in the proximal extracellular domain D4 (∼60%) (Liu et al., 1999). Remarkably, in almost all strains of *Cd4*^−/−^ mice that have been generated (mainly Cd4tm1Mak), *Cd4* deletion was achieved by targeting exon 5 (Killeen et al., 1993; Locksley et al., 1993; Mak et al., 1992; McCarrick et al., 1993; Rahemtulla et al., 1991; Rahemtulla et al., 1993), which would not disrupt expression of the alternative isoform. Moreover, an early study showed the existence of a natural alternative transcript in mouse brain tissue, with a start site located in exon 6, which was suggested to encode a shorter protein isoform (Gorman et al., 1987). In silico studies using the FANTOM5 CAGE-seq resource (Arner et al., 2015) through the ZENBU portal (Severin et al., 2014) confirmed the expression of this alternative transcript in adult mice corpus striatus and neonate mice diencephalon (Fig. S1 G). In humans, one transcript (NCBI Refseq variant 2: NM_001195014) is predicted to encode an isoform termed B0AZV7 (UniProt reference; 279 AA; Fig. S1 F) whereas the three other transcripts (NCBI Refseq variants 3–5: NM_001195015, NM_001195016, NM_001195017) are predicted to encode the B4DT49 isoform (Uniprot reference; 185 AA). Both isoforms are predicted to retain intact extracellular D4 and transmembrane and intracellular domains (Fig. 1 B). According to the UniProt database (https://www.uniprot.org/), CD4 (P01730) is experimentally shown to be expressed at the protein level. However, whether alternative transcripts (B0AZV7, B4DT49) are translated into protein isoforms of CD4 remains unknown. In silico studies show that CD4 (P01730) and isoform B4DT49 (referred to as isoform 2 from this point) have a signal peptide and therefore could be exported and expressed at the cell membrane (Fig. S1 H). Strikingly, the variants identified in P1–P6 were predicted to only affect the coding region of transcript variant 1 and hence expression of CD4 per se (Fig. 1 B). Indeed, *CD4* variants in P1 (c.491delA), P2–P3 (c.245C<G), and P4–P5 (c.235C<T) are in exons 4 and 5, which are only coding for transcript variant 1 (5′ untranslated region [UTR] for transcript variants 2–5) whereas the variant in P6 (c.1A>G) is in exon 2, which is spliced out (together with exon 3) for transcript variants 2–5. Note that variants detected in P7 are predicted to impact all transcripts. These in silico evidence led us to further investigate the consequences of *CD4* variants identified in the patients on mRNA and protein expression.

### Characterization of *CD4* mRNA transcripts in leukocytes

**Figure 2.**
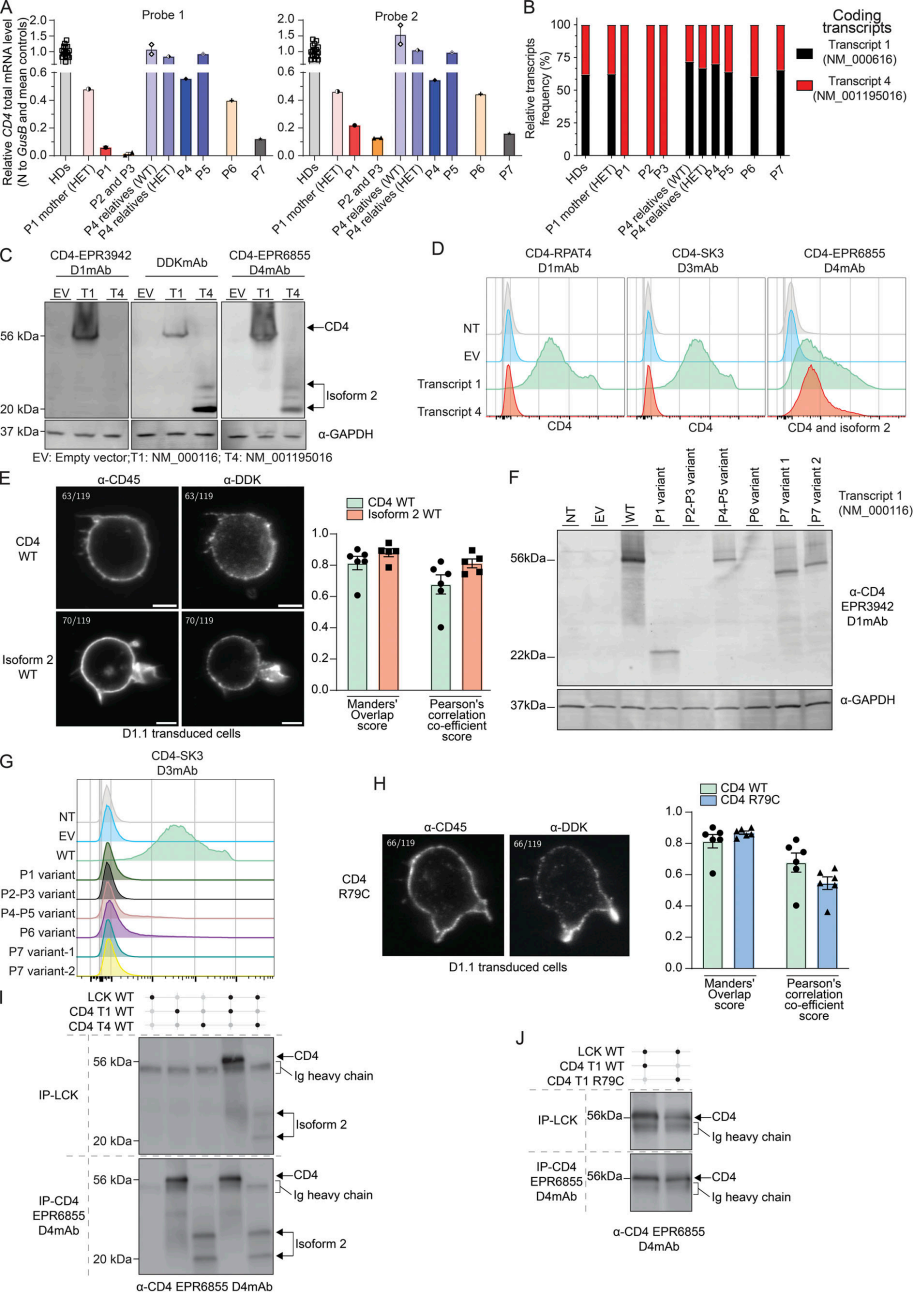
**Characterization of *CD4* transcripts and encoded proteins. (A)** RT-qPCR for total *CD4* mRNA (PBMCs) with two different probes (see Fig. 1 B). Bar represents the mean and the SD. Healthy donors (HDs, *n* = 18), P4 relatives (*n* = 2). **(B)** Transcripts relative abundance in cryopreserved PBMCs from HDs (*n* = 18), heterozygous (HET, *n* = 3), or WT relative (*n* = 1) and patients (P1–P7). **(C, D, F, G, I, and J)** HEK293T either non-transfected (NT) or transiently transfected with an empty vector (EV) or with vectors encoding the indicated CD4 transcript. **(E and H)** D1.1 Jurkat cells electroporated with vectors encoding indicated CD4 transcript. **(C)** Immunoblotting with (left) CD4 D1mAb (EPR3942), (middle) DDK mAb and (right) CD4 D4mAb (EPR6855) and GAPDH. T1: NM_000116; T2; NM_001195016. **(D)** Flow cytometry following extracellular staining of transfected HEK293T cells. Cells were stained with either (left) CD4 D1mAb (RPAT4), (middle) CD4 D3mAb (SK3), or (right) CD4 D4mAb (EPR6855). **(E)** Electroporated D1.1 cell slice acquired on Lattice Lightsheet microscope following anti-CD45 extracellular and anti-DDK intracellular labeling. Colocalization between CD4 (DDK) and cell surface (CD45) was assessed with Manders’ overlap score and Pearson’s correlation coefficient score. Bar represents the mean and the SD. Green: CD4/membrane overlap (*n* = 6); red: isoform 2/membrane overlap (*n* = 5). **(F)** Immunoblotting with N-terminal CD4 D1mAb (EPR3942) and GAPDH. **(G)** Flow cytometry following extracellular staining of transfected HEK293T cells. Cells were stained with CD4 D3mAb (SK3). **(H)** Electroporated D1.1 cell slice acquired on Lattice Lightsheet microscope following anti-CD45 extracellular and anti-DDK intracellular labeling. Colocalization between CD4 (DDK) and cell surface (CD45) was assessed with Manders’ overlap score and Pearson’s correlation coefficient score. Bar represents the mean and the SD. Green: CD4 WT/membrane overlap (*n* = 6); blue: CD4 R79C/membrane overlap (*n* = 6). **(I and J)** Immunoblots with CD4 D4mAb (EPR6855) HRP conjugated. Top: CD4 co-IP with LCK mAb. Bottom: direct-IP with CD4 D4mAb. Data are representative of at least two independent experiments. Source data are available for this figure: SourceData F2.

We first analyzed expression of *CD4* mRNA transcripts in cryopreserved total peripheral blood mononuclear cells (PBMCs) isolated from healthy donors. Two reverse transcription-quantitative polymerase chain reaction (RT-qPCR) probes were designed: Probe 1 exclusively detects transcript 1 (NM_000616, spans exons 2–3 junction) while Probe 2 detects all transcripts (span exons 6–7) (Fig. 1 B, blue line). Both probes detected *CD4* mRNA at comparable levels in PBMCs from healthy donors (ΔΔCT average 1.06 [0.76–1.55]) (Fig. S1 I). No signal for *CD4* mRNA was detected in sorted CD8^+^ T cells from healthy donors (data not shown). We then characterized the relative abundance of *CD4* transcripts in PBMCs from healthy donors by cloning, sequencing, and quantifying PCR-amplified full-length *CD4* cDNA. Transcript 1 was the predominant transcript expressed in healthy donors' PBMCs (∼62.1% [49.8–90.1], Fig. 2 B, black). Transcript 4 was also detected comprising ∼37.9% (9.8–50.1) of all *CD4* transcripts (Fig. 2 B, red), while other transcripts were not detected. We next analyzed PBMCs from all patients and some heterozygous family members. In patients with a frameshift deletion (P1) or nonsense variant (P2 and P3), *CD4* mRNA levels as assessed using Probe 1 were reduced by >90% and >99%, respectively (Fig. 2 A, left panel). In contrast, Probe 2 detected low but discernible levels of *CD4* mRNA in patient PBMC compared with levels measured for healthy donors (∼15–20%; Fig. 2 A, right panel). These data suggested *CD4* transcript 1 underwent nonsense-mediated mRNA decay in PBMCs from P1–P3. Consistent with these findings, cloning analysis exclusively detected transcript 4, which encodes isoform 2 in PBMCs from P1–P3 (Fig. 2 B, red). Unlike patients P1–P3, *CD4* mRNA levels were unaffected (P5) or reduced by 40% (P4), 60% (P6), and 85% (P7) relative to healthy donors (Fig. 2 A). In addition, the relative abundance of *CD4* transcripts in PBMCs from P4–P7 was comparable overall with healthy donors (Fig. 2 B). Transcript 2 (encoding B0AZV7) was not detected in any samples tested. Consistent with in silico predictions, *CD4* transcript 4 was invariably found to be WT in P1–P6, whereas the splice-site variant identified in P7 affected both transcripts 1 and 4. Overall, PBMCs from patients with missense (P4–P6) or essential splice site (P7) variants expressed both *CD4* transcripts 1 and 4 at levels similar (P5) or reduced (P4, P6, P7) compared with healthy donors, whereas cells from patients with a frameshift deletion (P1) or a nonsense variant (P2, P3) expressed greatly reduced but nonetheless detectable levels of *CD4* mRNA comprising exclusively of WT transcript 4 (encoding isoform 2). These observations led us to investigate WT and mutant CD4 and isoform 2 expression.

### Characterization of CD4 protein and isoform 2 in overexpression system

We assessed CD4 and isoform 2 proteins by overexpression of relevant C-terminal DDK–tagged vectors in HEK293T cells and analysis by western blot and flow cytometry using four different mAbs: (1) against the DDK epitope tag (clone D6W5B, DDK mAb); (2) anti-CD4 domain 1 (clones RPAT4 and EPR3942, D1mAb); (3) anti-CD4 domain 3 (clone SK3, D3mAb); and (4) anti-CD4 domain 4 (EPR6855, D4mAb) (Fig. 1 C). First, we showed that CD4 D1mAb (EPR3942) could only detect CD4 in the overexpression system by western blotting, while DDK mAb and CD4 D4mAb detected both CD4 and isoform 2 at the expected molecular weight (MW) (Fig. 2 C). Flow cytometry analysis following extracellular staining with D1 (RPAT4), D3, or D4 specific mAbs revealed that CD4 and isoforms 2 can be expressed at the membrane of transfected HEK293T cells (Fig. 2 D). Double staining with mAbs specific for D1 (RPAT4) and D3 (SK3), or D3 (SK3) and D4 (EPR6855) confirmed the specificity of these mAbs (data not shown). Surface expression of WT CD4 and isoform 2 were also confirmed by Lattice Lightsheet microscopy using anti-CD45 mAb extracellular staining to label the membrane and anti-DDK intracellular staining to detect CD4 and isoform 2 in electroporated D1.1 T cell line to assess colocalization (Fig. 2 E). Next, we assessed the impact of *CD4* variants on CD4 expression in our overexpression system. Western blot and extracellular staining followed by flow cytometric analysis of transiently transfected HEK293T cells revealed that *CD4* variants identified in public databases did not affect CD4 protein expression (Fig. S1, J and K). Q164Rfs*34 cDNA (Kindred A, P1) encoded a truncated protein (22 kD, Fig. S1 C) that could be detected with the N-terminal CD4 D1mAb (EPR3942) (Fig. 2 F). No expression of the S82* (Kindred B; P2, P3) CD4 protein was detected, while CD4 R79C (Kindred C; P4, P5) was expressed as a full-length protein (Fig. 2 F). Both V386_R426delfs*13 and p.V386_Q395delfs*45 CD4 protein (P7 variants 1 and 2) were expressed and detected at reduced MW as expected (Fig. 2 F). However, despite similar transfection efficiencies (Fig. S1 L), none of the cDNAs encoding *CD4* variants identified in the patients (P1–P7) yielded expression of a protein that could be detected by extracellular surface staining and flow cytometric analysis of transfected HEK293T cells using the CD4 D1 or D3mAb (Fig. 2 G and Fig. S1 M). Nonetheless, surface expression of CD4 R79C (P4–P5) was similar to CD4 WT (Manders’ overlap score mean >0.8 and Pearson’s correlation co-efficient score mean >0.5) by microscopy using anti-CD45 extracellular staining and anti-DDK intracellular staining in D1.1 electroporated T cell line (Fig. 2 H). These data suggest that rather than impacting intracellular trafficking, the missense R79C variant in P4 and P5 affected protein folding and therefore the 3D structure of CD4, with a cysteine replacing the arginine at position 79. This likely prevents detection of CD4 surface expression by mAbs against distal extracellular domains (D1 and D3mAbs) by flow cytometry, but not by western blot in denaturing condition (D1mAb). Overall, we established that, unlike variants present in public databases, variants identified in *CD4* in P1–P3, P6, and P7 abolished cell surface expression of CD4, while the variant in P4 and P5 likely affected protein conformation, preventing detection of the mutated CD4 by D1 or D3mAbs. Moreover, these data establish that, like WT CD4, isoform 2 can also be expressed at the cell surface, at least when overexpressed in vitro.

### Both CD4 and isoform 2 can interact with LCK

The cytoplasmic domain of CD4 associates with the protein tyrosine kinase LCK, which enhances intracellular signaling initiated following engagement of the TCR by peptide/MHC class II complexes on APCs (Glassman et al., 2018; Li et al., 2017; Mingueneau et al., 2008; Xu and Littman, 1993). Thus, by performing immunoprecipitation (IP) on whole cell lysates prepared from transiently transfected HEK293T cells, we investigated whether the different isoforms encoded by human *CD4* can interact with LCK. As expected, WT CD4 and isoform 2 were detected by direct IP with anti-CD4 D4mAb (Fig. 2 I, bottom panel, lines 2–5). More importantly, both proteins were also detected after co-IP with anti-LCK mAb (Fig. 2 I, top panel, lines 4–5). These data indicate that isoform 2, which can be expressed on the cell surface (Fig. 2, D and F), retains the ability to interact with LCK.

Furthermore, despite its altered conformation, CD4 R79C (P4–P5) was found to be expressed on the surface of transfected T cell lines. Therefore, we assessed its ability to interact with LCK. WT or R79C CD4 were detected both by direct IP with anti-CD4 D4mAb (Fig. 2 J, bottom panel), but more importantly also after co-IP with anti-LCK mAb and western blotting with anti-CD4 D4mAb (Fig. 2 J, top panel). These data indicate that CD4 R79C (P4–P5) has a preserved capacity to interact with LCK. Thus, CD4 R79C or WT isoform 2 expressed at the cell surface with an intact intracellular domain retain their ability to interact with LCK in vitro.

### Expression of CD4 and isoform 2 by CD3^+^TCRαβ^+^CD8^−^ T cells

**Figure 3.**
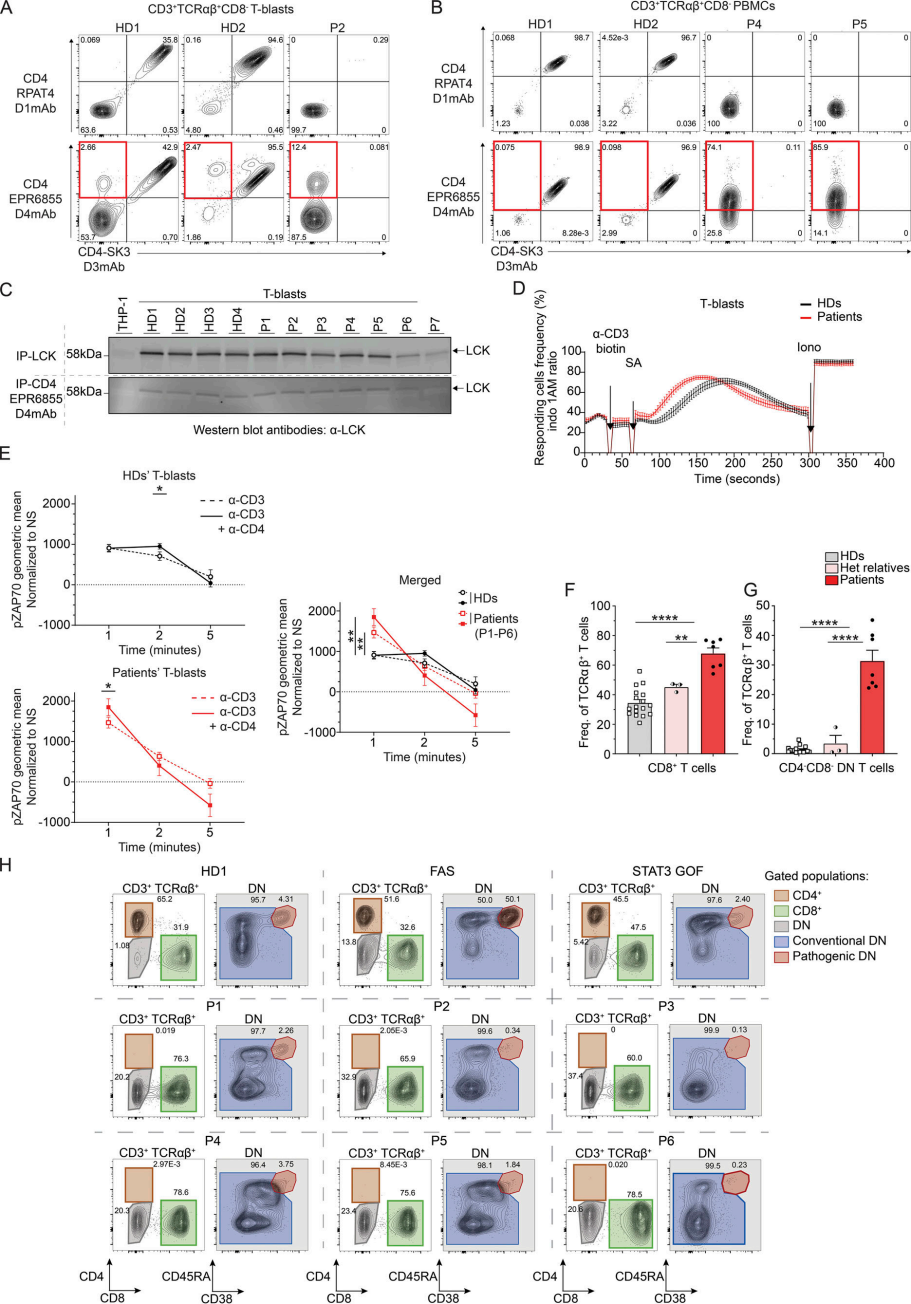
**Characterization of the T cell compartment in patients with CD4 deficiency. (A)** Flow cytometry following extracellular staining of T-blasts from two healthy donors (HDs) and P2. Cells were gated as follows: CD20^−^CD3^+^TCRγδ^−^TCRαβ^+^CD8^−^. Y axis represents (top) CD4 D1mAb (RPAT4) or (bottom) CD4 D4mAb (EPR6855). X axis represents CD4 D3mAb (SK3). Red gate represents the cell population expressing isoform 2 only. **(B)** Flow cytometry following extracellular staining of PBMCs from two HDs, P4, and P5. Cells were gated as follows: CD20^−^CD3^+^TCRγδ^−^TCRαβ^+^CD8^−^. Y axis represents (top) CD4 D1mAb (RPAT4) or (bottom) CD4 D4 mAb (EPR6855). X axis represents CD4 D3mAb (SK3). Red gate represents the CD4 D4mAb single positive cell population. **(C)** Immunoblots with LCK and GAPDH. Top: direct-IP with LCK; bottom: LCK co-IP with CD4 D4mAb (EPR6855). Experiments were conducted in THP1, T-blasts from four healthy donors (HD1–HD4) and patients (P1–P7). **(D)** Calcium flux mobilization in T-blasts from HDs (black, *n* = 7) and patients (red, P1–P6) upon TCR activation assessed by flow cytometry. Y axis represents the frequency of responding cells (ratio indo 1AM). X axis represents the time of acquisition. Arrows represent stimulation. SA: streptavidin; Iono: ionomycin. Each data point represents an average of 3 s. **(E)** ZAP70 phosphorylation after crosslink. Rested T-blasts from HDs (*n* = 8, black) and patients (P1–P6, red), incubated in the absence or presence of either biotinylated CD3 (OKT3) alone (plain line) or CD3 (OKT3) with CD4 (D4mAb) (dotted line), and crosslinked with streptavidin for 1, 2, and 5 min. The geometric mean of ZAP70 phosphorylation was assessed by flowcytometry. Y axis: geometric mean of ZAP70 phosphorylation upon stimulation normalized by non-stimulated condition; x axis: time of stimulation (streptavidin only). Statistical analysis by one-tailed parametric paired Student’s *t* test. *P < 0.05; **P < 0.01. **(F)** CD3^+^TCRαβ^+^ CD8^−^ and CD8^+^ T cell frequencies in HDs (*n* = 18), heterozygous relatives (*n* = 3), and patients (P1–P6). **(G)** CD3^+^TCRαβ^+^CD8^−^CD4^−^ (CD4 D3mAB) DN T cell frequencies in HDs (*n* = 18), heterozygous relatives (*n* = 3), and patients (P1–P6). **(F and G)** Statistical analysis by one-way ANOVA with multiple comparisons (Tukey). **P < 0.01; ****P < 0.0001. **(H)** CD38 and CD45 staining defining CD3^+^TCRαβ^+^ conventional DN (CD38^−^CD45^+/−^ blue gate) and pathogenic (CD38^−^CD45^+/−^, red gate) cell frequencies in one representative HD, FAS-deficient patient, STAT3 GOF, and CD4 patients with *CD4* mutations (P1–P6). Brown gates represent total CD4^+^ T cells, green gates represent total CD8^+^ T cells, gray gates represent total DN cells. Data are representative of at least two independent experiments. Source data are available for this figure: SourceData F3.

**Figure S2.**
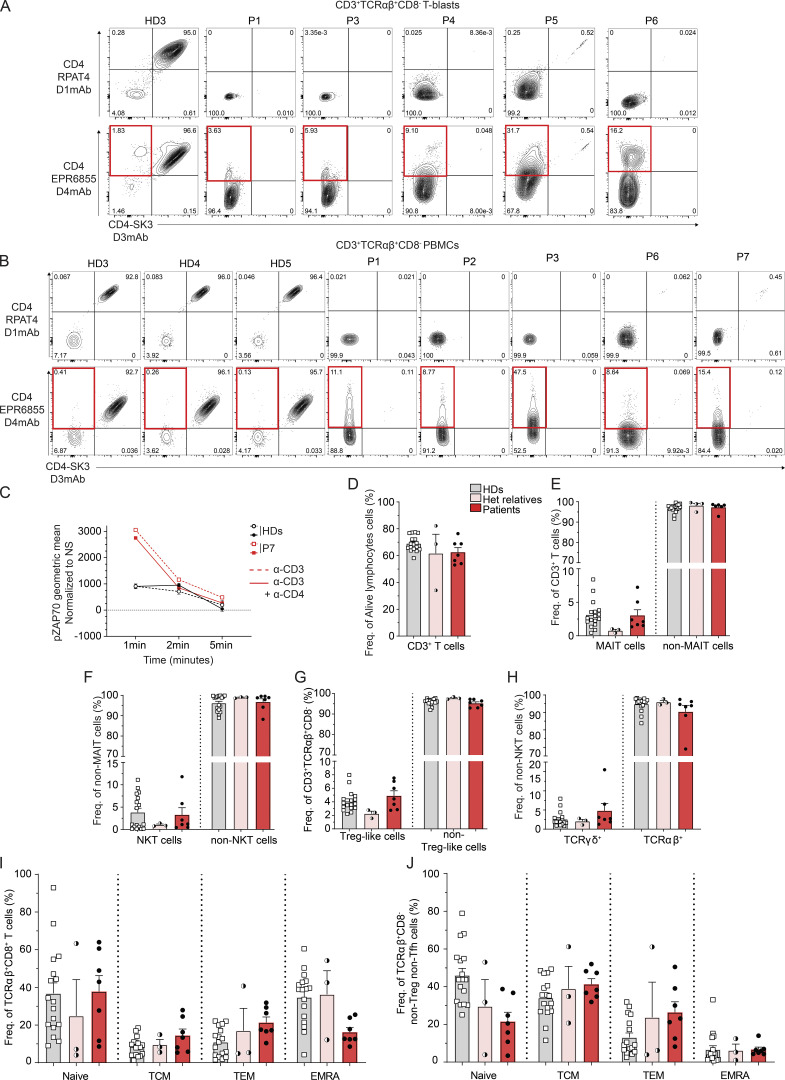
**Impact of *CD4* variants on leukocyte differentiation, function, and TCR signaling. (A)** Flow cytometry following extracellular staining of T-blasts from one healthy donor (HD3) and patients (P1, P3–P6). Cells were gated as follows: CD20^−^CD3^+^TCRγδ^−^TCRαβ^+^CD8^−^. Y axis represents (top) CD4 D1mAb (RPAT4) or (bottom) CD4 D4mAb (EPR6855). X axis represents CD4 D3mAb (SK3). Red gate represents the CD4 D4mAb single positive cell population. **(B)** Flow cytometry following extracellular staining of cryopreserved PBMCs from three HDs and patients (P1–P3, P6, P7). Cells were gated as follows: CD20^−^CD3^+^TCRγδ^−^TCRαβ^+^CD8^−^. Y axis represents (top) CD4 D1mAb (RPAT4) or (bottom) CD4 D4 mAb (EPR6855). X axis represents CD4 D3mAb (SK3). Red gate represents the CD4 D4mAb single positive cell population. **(C)** ZAP70 phosphorylation after crosslink. Rested T-blasts from P7, incubated in the absence or presence of either biotinylated CD3 (OKT3) alone (plain line) or CD3 (OKT3) with CD4 (D4mAb) (dotted line), and crosslinked with streptavidin for 1, 2, and 5 min. The geometric mean of ZAP70 phosphorylation was assessed by flowcytometry. Y axis: the geometric mean of ZAP70 phosphorylation upon stimulation normalized by non-stimulated condition; x axis: time of stimulation (streptavidin only). **(D)** CD3^+^ T lymphocytes subset frequencies in HDs, heterozygous relatives, and patients (P1–P7). **(E)** MAIT (CD3^+^CD161^+^TCR-Vα7.2^+^) cell frequency in HDs, heterozygous relatives, and patients (P1–P7). **(F)** NKT cell (CD3^+^Vα24JαQ^+^non-MAIT) frequency in HDs, heterozygous relatives, and patients (P1–P7). **(G)** CD3^+^TCRαβ^+^CD8^−^CD127^−^CD25^+^) Treg-like cell frequencies in HDs, heterozygous relatives, and patients (P1–P7). **(H)** CD3^+^ TCRγδ^+^ and TCRαβ^+^ frequencies in HDs, heterozygous relatives, and patients (P1–P7). **(I and J)** CD3^+^TCRαβ^+^CD8^+^ (I) and CD3^+^TCRαβ^+^CD8^−^ (J) T cell subpopulation frequencies in HDs, heterozygous relatives and patients (P1–P7). TCM: T central memory; TEM: T effector memory; EMRA: CD45RA^+^ effector memory. Data are representative of at least two independent experiments.

Based on the above findings, we proceeded to determine endogenous expression of CD4 and isoform 2 by extracellular staining and flow cytometric analysis of PBMCs and activated T cells (T-blasts) generated from healthy donors’ and patients’ PBMCs (P1–P7). CD3^+^TCRαβ^+^CD8^−^ T-blasts expressing surface CD4 (CD4 D1mAb/D3mAb or D4mAb/D3mAb double positive; C1–C3, Fig. 3 A and Fig. S2 A) or isoform 2 (D4mAb single positive, red square) were clearly detectable in healthy donors. In PBMCs from healthy donors, <0.5% of CD3^+^TCRαβ^+^CD8^−^ T cells reacted with the D4mAb only (C1–C5, red square, Fig. 3 B and Fig. S2 B).

In contrast to healthy donors, no surface expression of CD4 was detected by D1 or D3mAb on CD3^+^TCRαβ^+^CD8^−^ T-blasts generated from the patients (Fig. 3 A and Fig. S2 A, top panels) or on CD3^+^TCRαβ^+^CD8^−^ T cells present in PBMCs (P1–P7, Fig. 3 B and Fig. S2 B top panels). However, surface expression of mutant CD4 (P4, P5), WT (P1–P6), or mutant (P7) isoform 2 was observed with D4mAb on patients’ T-blasts (Fig. 3 A and Fig. S2 A, red squares) as well as PBMCs (P1: 11.1%; P2: 8.77%; P3: 4.33%; P4: 74.1%; P5: 85.9%; P6: 8.64%, P7: 15.4%) (Fig. 3 B and Fig. S2 B, red squares). It is important to note that in cells from P4 and P5, because D4mAb can detect both CD4 R79C and WT isoform 2 expression, this system does not allow isoform discrimination. These findings establish that T cells from patients harboring biallelic *CD4* variants express protein encoded by the *CD4* gene at the cell surface, prompting us to further investigate the role of patient *CD4* alleles in human T cells.

### CD4 and isoform 2 interact with LCK in activated T cells

Having established that WT CD4, R79C CD4, and WT isoform 2 could interact with LCK when overexpressed in HEK293T cells, we wanted to determine whether this also occurred in primary T-blasts from healthy donors and patients. As expected, in healthy donors' T-blasts, LCK was detected in immunoprecipitates captured by anti-LCK mAb (Fig. 3 C, top panel, lanes C1–C4), as well as by anti-CD4 D4mAb (Fig. 3 C, bottom panel, lanes C1–C4). Importantly, no LCK was detected in immunoprecipitates from the human myeloid cell line THP1, which expresses CD4 but not LCK and was used as a negative control (Fig. 3 C, lane THP-1).

In T-blasts generated from patients expressing CD4 R79C or isoform 2 WT with intact intracellular domains (P1–P6), LCK was detected by direct IP with anti-LCK mAb (Fig. 3 C, top panel, lane P1–P6), as well as after co-IP with anti-CD4 D4mAb and detection by anti-LCK mAb (Fig. 3 C, bottom panel, lanes P1–P6). However, no LCK was detected after co-IP with anti-CD4 D4mAb in T-blasts from P7 (Fig. 3 C, bottom panel, lane P7). This is consistent with the *CD4* variant identified in P7 (c.1157-1 G>A) being predicted to truncate the intracellular region of both CD4 and isoform 2, which contains binding sites for LCK. Overall, these findings establish that endogenous protein encoded by *CD4* with intact intracellular sequences and expressed on the surface of patients’ T cells (P1–P6) retains the ability to interact with LCK similarly to WT CD4 expressed by healthy donors’ T cells.

### Intact calcium mobilization upon TCR activation in patient-activated T cells

We next characterized TCR signaling in healthy donor and patient T cells by measuring intracellular calcium mobilization in a fluorescent-based assay. Upon TCR activation with biotinylated anti-CD3 mAb crosslinked by streptavidin, we observed an increase in calcium released into the cytoplasm of CD3^+^CD8^−^ T-blasts from healthy donors (maximum [max] >70% of responding cells 150 s after adding streptavidin). An increased calcium release was also observed upon TCR ligation on CD3^+^CD8^−^ T-blasts generated from patients (P1–P7) (max >70% of responding cells 120 s after adding streptavidin). The magnitude of the calcium flux exhibited by T cells from healthy donors and patients in response to anti-CD3 mAb, or ionomycin used as a positive control (>88% of responding cells), were comparable (Fig. 3 D). Thus, TCR signaling in patient (P1–P7) CD3^+^CD8^−^ T cells is intact upon CD3 engagement. This led us to further investigate the role of the different patients’ endogenous isoforms in TCR signaling.

### CD4 and isoform 2 function in TCR signaling

Despite detectable surface expression and interaction with LCK, isoform 2 lacks the three distal extracellular domains, including D1 and D2 which, unlike D4 domain, are critical for interactions between HLA class II and CD4 (Clayton et al., 1989; Fleury et al., 1991). Similarly, the distal extracellular domains of CD4 R79C (P4 and P5) appears to have disrupted folding and 3D conformation. Consequently, these isoforms are unlikely to be recruited to the TCR/CD3-HLA class II macrocomplex via direct interactions with HLA class II (Glassman et al., 2018). However, reciprocal binding between membrane proximal domains of CD4 and CD3 subunits has been described (Glassman et al., 2018; Lynch et al., 1999; Nakamura et al., 2003; Vignali et al., 1996; Wu et al., 1997) as well as through intracellular interactions involving recruited LCK (Li et al., 2017; Mingueneau et al., 2008; Xu and Littman, 1993). Upon CD4 recruitment to the macrocomplex, associated LCK phosphorylates ZAP70 which then activates its substrates, allowing downstream TCR signal transduction (Au-Yeung et al., 2018). To further characterize TCR signaling in the presence of altered CD4 expression, we first evaluated the ability of CD4 to enhance TCR proximal signaling in healthy donors’ T-blasts upon CD3/CD4 crosslinking by assessing phosphorylation of ZAP70.

T-blasts from healthy donors were incubated with either biotinylated anti-CD3 (OKT3) mAb alone or together with anti-CD4 (D4mAb), followed by crosslinking with streptavidin for 1, 2, or 5 min, after which time ZAP70 phosphorylation was assessed by flow cytometry. We observed a similar increase of ZAP70 phosphorylation upon crosslinking CD3 alone or with CD4 D4mAb at 1 min in T-blasts from healthy donors (Fig. 3 E, top left panel). However, after 2 min of crosslinking, the level of ZAP70 phosphorylation was significantly higher in T-blasts stimulated via CD3/CD4 compared with CD3 alone (Fig. 3 E, top left panel). This suggests that WT CD4 sustains TCR signaling when recruited to TCR-CD3 macrocomplex in T cells from healthy donors.

In rested T-blasts from all patients expressing endogenous protein encoded by *CD4* capable of interacting with LCK (P1–P6), phosphorylation of ZAP70 induced via CD3/CD4 crosslinking at 1 min was significantly higher than that achieved by anti-CD3 mAb alone (Fig. 3 E, bottom left panel). This was not observed in T-blasts generated from P7 (Fig. S2 C) due to the inability of mutant CD4 or isoform 2 expressed by this patient’s cells to recruit LCK. At 2 and 5 min after activation, ZAP70 phosphorylation decreased for all crosslinking conditions. Interestingly, ZAP70 phosphorylation was significantly higher in patients’ T-blasts compared with healthy donors' T-blasts at 1 min, which was not the case at later time points (Fig. 3 E, right panel). Together, these data show additive effects of engaging CD3 and CD4 R79C or isoform 2 WT on ZAP70 phosphorylation in patients’ T-blasts, suggesting patients’ isoforms are functional and enhance TCR signaling despite a different kinetic and magnitude than WT CD4.

### Immunophenotype of patients’ T cells

Immunophenotyping of all patients’ blood cells (P1–P7) revealed normal numbers of total CD3^+^ T cells, normal or elevated numbers of CD3^+^CD8^+^ T cells, but a complete absence of CD3^+^CD4^+^ T cells (Table S2 and Table S3). We confirmed these data by in-depth flow cytometric analysis to determine the impact of *CD4* variants on the development and differentiation of circulating T cells in all patients. Overall frequencies of CD3^+^ T cells were similar (P1, P5, P6) or slightly decreased (P2–P4, P7) in patients compared with healthy donors (Fig. S2 D). Within the population of CD3^+^ T cells, frequencies of mucosal-associated invariant T (MAIT) cells (CD3^+^CD161^+^TCR-Vα7.2^+^), natural killer (NK) T cells (Vα24JαQ^+^), and regulatory T (Treg)–like cells (CD8^−^CD127^−^CD25^+^) were also comparable between patients and healthy donors (Fig. S2, E–G). Frequencies of TCRαβ^+^ and TCRγδ^+^ T cells were similar (P1–P6) or increased (P7) compared with healthy donors (Fig. S2 H). Despite normal T cell frequencies, all patients had significant and dramatically increased proportions of CD8^+^ T cells (patients: mean 67.8% [range 54.1–77.4%], healthy donors: mean 34.5% [range: 20.9–55.8%]) (Fig. 3 F), resulting in a skewed CD8^+^/CD8^−^ ratio in the TCRαβ^+^ compartment of the patients (∼1.5) compared with healthy donors (∼0.65). Using conventional flow cytometry mAbs against human CD4 (D3), a significant increase in frequency of TCRαβ^+^ CD4^−^CD8^−^ double negative (DN) T cells was observed in all patients (mean: 31.3%; P1: 38.7%, P2: 26.6%, P3: 40.1%, P4: 22.2%, P5: 22.8%, P6: 24%, P7: 45.2%) compared with healthy donors (mean: 1.5% [range 0.6–4.7%], Fig. 3 G). Frequencies of CD8^−^ and CD8^+^ T cell subsets in the patients were largely within the range for healthy donors, except for CD8^+^CD45RA^+^ effector memory cells, which were low in all patients (P1–P7), while proportions of CD8^−^ and CD8^+^ T naive cells were strikingly decreased, and memory cells increased in P2 and P3 compared with healthy donors (Fig. S2, I and J). Overall, the most remarkable T cell phenotypes in patients with damaging *CD4* variants were the skewed CD8^+^/CD8^−^ ratio and the expansion of TCRαβ^+^ DN T cell population.

### Expanded TCRαβ^+^ CD4^−^CD8^−^ DN T cells in patients have a conventional rather than pathogenic phenotype

While DN T cells can be detected at low frequencies in peripheral blood of healthy individuals (Fig. 3 G), they are however expanded and pathognomonic of diseases of immune dysregulation such as autoimmune lymphoproliferative syndrome (ALPS) due to FAS deficiency (Seif et al., 2010), PD-1 deficiency (Ogishi et al., 2021), or STAT3 gain of function (GOF) (Nabhani et al., 2017). To define the expanded population of DN T cells in inherited CD4 deficiency, we immunophenotyped these cells using a flow cytometric panel adapted from a recent study that characterized conventional DN (CD38^−^CD45^+/−^) versus pathogenic DN (CD38^+^CD45^+^) T cells in FAS-deficient patients (Maccari et al., 2021). In our hands, proportions of CD3^+^TCRαβ^+^ DN cells in CD4-deficient patients (mean: 25.8% [range: 20.2–37.4%]), FAS deficiency (13.8%), and STAT3 GOF (5.4%) were greatly increased compared with healthy donors (1.08%) (Fig. 3 H). Furthermore, the DN T cell population in FAS deficiency is comprised of almost equal proportions of conventional and pathogenic DN cells. In contrast, the DN T cell population in healthy donors, CD4-deficient patients (*n* = 6), and STAT3 GOF patients was comprised predominantly of conventional DN cells (healthy donor: 95.7%; CD4-deficient patients: 98.5%; STAT3 GOF: 97.6%; Fig. 3 H). Thus, the expanded population of TCRαβ^+^ DN cells in CD4 deficiency does not correspond to the pathogenic DN subset typical of FAS deficiency.

### Molecular characterization of the TCR repertoire of patients’ TCRαβ^+^ CD8^−^ T cells

**Figure S3.**
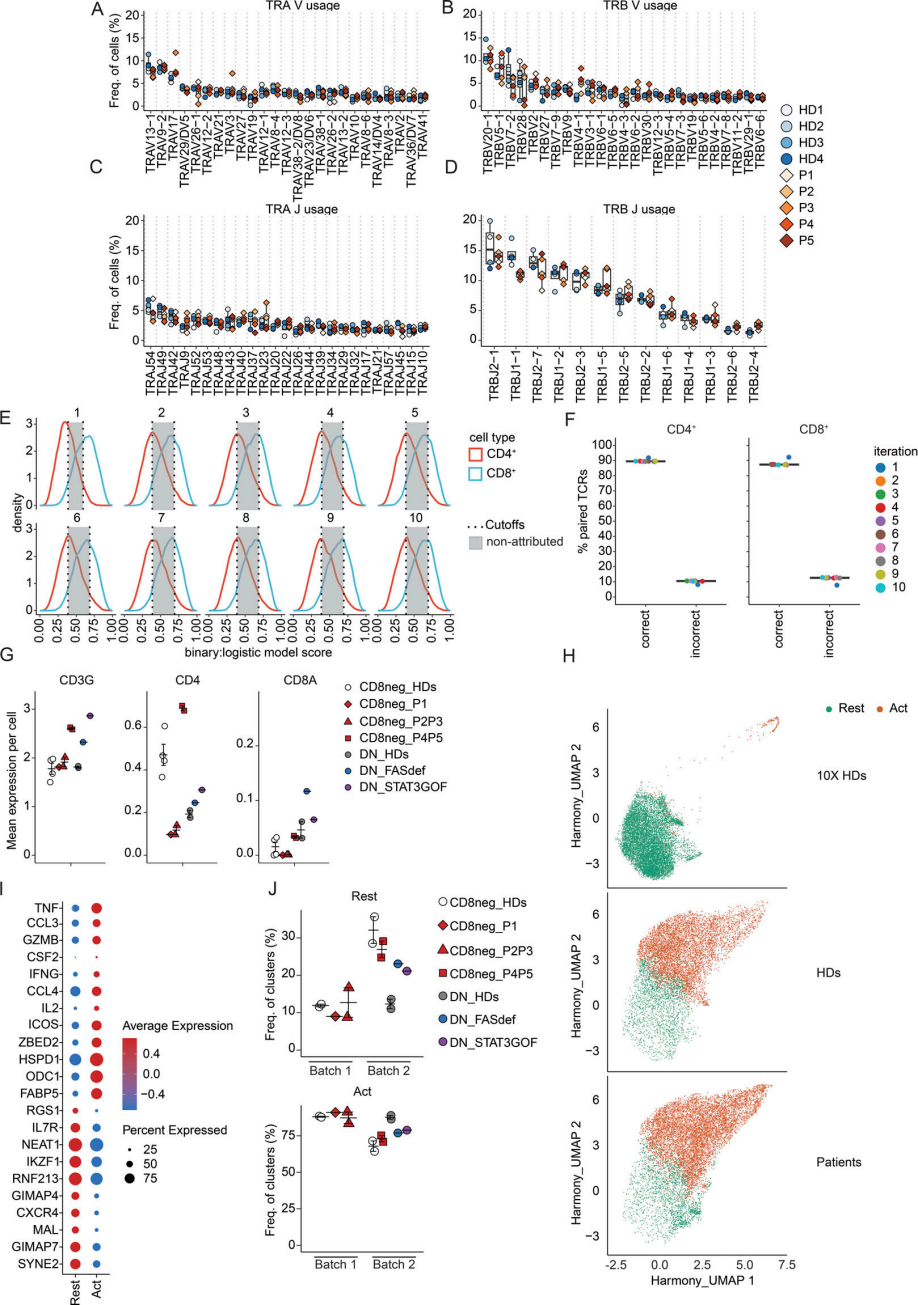
**Short- and long-read scRNAseq analysis of activated memory CD3**^**+**^**TCRαβ**^**+**^**CD8**^**−**^ **T cells. (A–D)** Diversity of the TCR repertoire (Vα, Vβ, Jα, and Jβ gene sequences) expressed by memory TCRαβ^+^CD8^−^ T cells in four healthy donors and five patients (P1–P5) by high-throughput targeted long-read single-cell sequencing. **(E)** 10 iterations generated as a training set with subsampled known CD4^+^ and CD8^+^ TCRαβ sequences from Carter data. Accuracy threshold to correctly predict CD4^+^ (true positive) and CD8^+^ (true positive) was set based on the fifth percentile for the CD8 distribution and the 95th percentile for the CD4 distribution. The red curve represents CD4^+^ predicted frequency, and the blue curve represents CD8^+^ predicted frequency. The gray area represents not attributed cells. **(F)** Prediction accuracy after removing not-attributed cells. **(G–I)** scRNAseq analysis of stimulated memory CD3^+^TCRαβ^+^CD8^−^ cells from four healthy donors (CD8neg_HDs) and five patients (CD8neg_P1–P5) as well as stimulated memory CD3^+^TCRαβ^+^ DN cells from two healthy donors (DN_HDs), FAS-deficient (DN_FASdef), and *STAT3* GOF (DN_STAT3GOF) patients. **(G)** Mean single-cell-level expression of *CD3G* (left), *CD4* (middle), and *CD8A* (right) mRNA. **(H)** UMAP plots showing resting (Rest, green) and activated (Act, orange) clusters. **(I)** Dot plot representing the top differentially expressed genes in resting versus activated clusters. **(J)** Proportion of resting and activated clusters in each sample. Data are representative of at least two independent experiments.

To better understand the nature of T cells present in patients with *CD4* mutations, we determined the diversity of the TCR repertoire expressed by memory TCRαβ^+^CD8^−^ T cells in healthy donors and the novel CD4-deficient patients (P1–P5) by high-throughput targeted long-read single-cell sequencing. Analysis of TCR Vα, Vβ, Jα, and Jβ gene sequences showed that the TCR repertoire of, and VJ gene usage by, memory TCRαβ^+^CD8^−^ T cells was similar in patients and healthy donors (Fig. S3, A–D). These data suggest that there is no preferential expansion of cells with restricted diversity in the TCRαβ^+^CD8^−^ T cell population in CD4-deficient patients.

**Figure 4.**
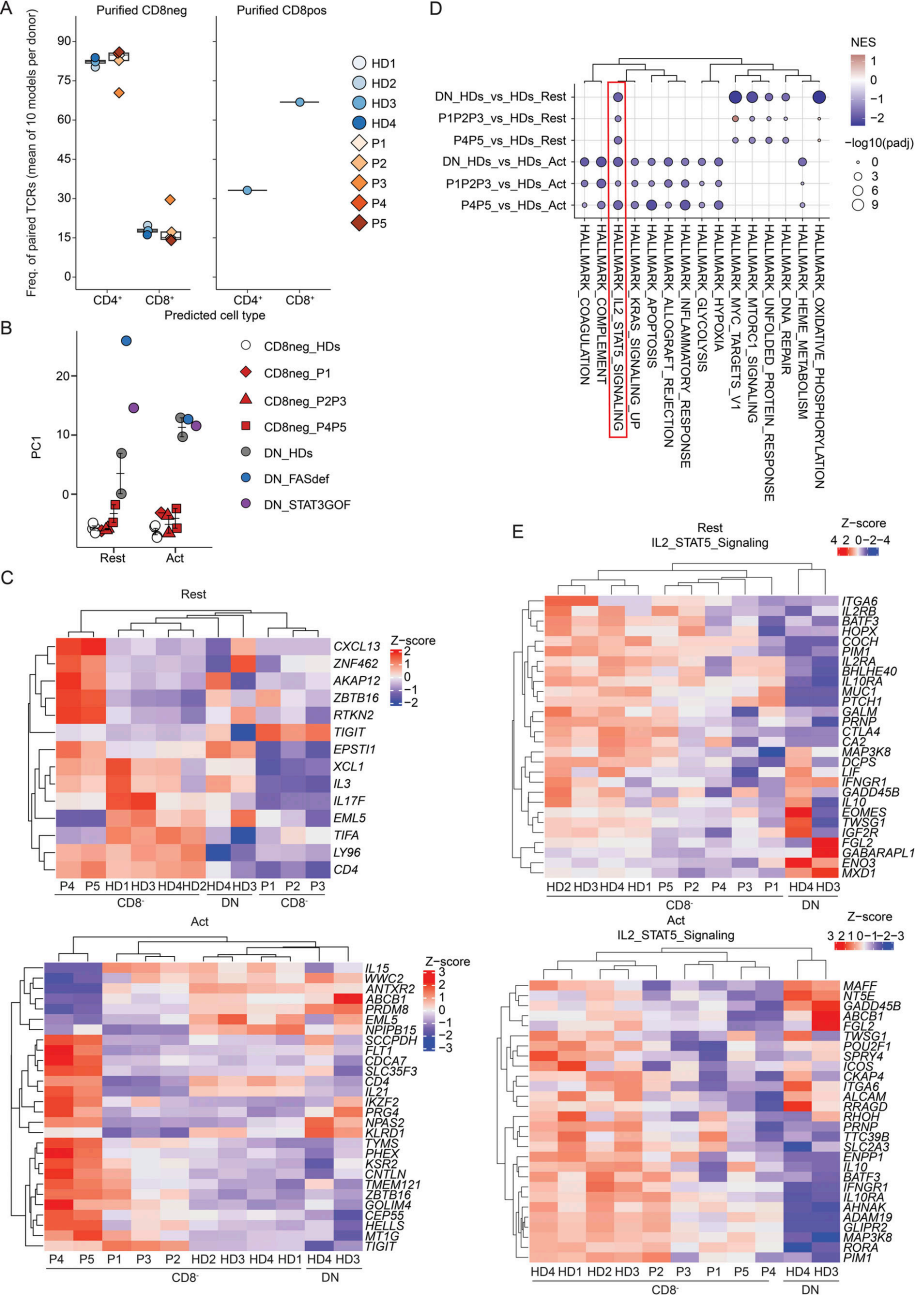
**Transcriptomic analysis of activated memory CD3**^**+**^**TCRαβ**^**+**^**CD8**^**−**^ **T cells. (A)** αβ sequences paired analysis following isolation, targeted capture, long-read sequencing, and bioinformatic sequence reconstruction at single nucleotide resolution. TCRαβ paired sequences from healthy donors (HDs, *n* = 4) and five CD4-deficient patients (P1–P5) were compared with public TCR sequence data. **(B–E)** scRNAseq analysis. Sorted memory CD3^+^TCRαβ^+^CD8^−^ cells were stimulated with anti-CD2/CD3/CD28 beads for 20 h. Data from healthy donors (*n* = 4) and five patients (P1–P5), obtained in two batches of experiments, were integrated together with non-stimulated PBMC datasets obtained from the 10x Genomics web portal. As a comparison, memory CD3^+^TCRαβ^+^CD4^−^CD8^−^ (DN) cells from two healthy donors (DN_HDs), FAS-deficient (DN_FASdef), and *STAT3* GOF (DN_STAT3GOF) patients were also integrated. Cells transcriptionally similar to regulatory T, γδ T, and MAIT cells were excluded from the subsequent analyses. **(B)** Principal component (PC) analysis of resting (left) or activated (right) population. **(C–E)** Pseudobulk differential expression analysis between memory CD3^+^TCRαβ^+^CD8^−^ cells from healthy donors (HD1–HD4_CD8neg) and patients (P1–P5_CD8neg). Patients with frameshift/nonsense variants (P1–P3) and missense variants (P4–P5) were separately compared to HDs. **(C)** Heatmap of significantly differentially expressed gene in patients compared to HDs (log_2_ fold change >1 or less-than −1; adjusted P value <10^−3^). Genes identified in either of the two comparisons (P1–P3_CD8neg versus HD1–HD4_CD8neg or P4–P5_CD8neg versus HD1–HD4_CD8neg) are shown. Results for (top) resting and (bottom) activated populations are shown separately. **(D)** Hallmark pathways found to be downregulated in stimulated memory CD3^+^TCRαβ^+^ DN cells from two healthy donors (DN_HDs) compared with stimulated memory CD3^+^TCRαβ^+^CD8^−^ cells from 4 HDs and patients (P1–P5). Y axis represents comparisons; x axis represents gene sets reordered based on the non-supervised hierarchical clustering. Jaccard distance was calculated between each gene set based on their gene composition. **(E)** Heatmap analysis of leading-edge genes for the Hallmark IL-2/STAT5 signaling pathway identified through GSEA. Genes recurrently identified in the two comparisons (P1–P3_CD8neg versus HD1–HD4_CD8neg and P4–P5_CD8neg versus HD1–HD4_CD8neg) are shown. Results for (top) resting and (bottom) activated populations are shown separately.

Additional bioinformatic analyses were performed to enable comparison of single-cell TCRα and TCRβ sequences from CD4-deficient patients to recently published TCR sequences derived from healthy donor CD4^+^ or CD8^+^ T cells (Carter sequences) (Carter et al., 2019). In this analysis, individual TCR sequences from five patients (P1–P5) and four healthy donors were compared with Carter sequences. To obtain greater resolution, only paired TCRαβ sequences were considered. 10 iterations were generated with subsampled known CD4^+^ and CD8^+^ TCR sequences from Carter data as the training set. For the 10 iterations, the accuracy threshold to correctly predict CD4^+^ (true positive) and CD8^+^ (true positive) T cells was set based on the fifth percentile for the CD8 distribution and the 95th percentile for the CD4 distribution (Fig. S3 E). After removing any cell assigned with a probability between the two cut-offs, the prediction accuracy was ∼90% (Fig. S3 F). More than 80% of TCRαβ^+^ CD8^−^ T cells from healthy donors (HD1–HD4; mean 82.2% [80.3–83.8]) could be confidently predicted to be CD4^+^ T cells based on their TCRαβ paired sequences (Fig. 4 A, left panel). Similarly, 60% of TCRαβ^+^ CD8^+^ T cells from one healthy donor (HD3) were predicted to be CD8^+^ T cells (Fig. 4 A, right panel). Strikingly, most TCRαβ^+^ CD8^−^ T cells from patients with *CD4* mutations were also confidently predicted (mean 81.9%, range [70.4–86%]) to be CD4^+^ T cells (Fig. 4 A, right panel). Overall, the TCRαβ^+^ repertoire and gene usage of CD8^−^ memory T cells from patients with *CD4* mutations resembled healthy donors. Furthermore, the majority of the phenotypically CD4^−^CD8^−^ T cells detected in the patients would be predicted to be CD4^+^ T cells based on their TCR sequences.

### Single-cell transcriptomic analysis of CD8^−^ T cells in CD4-deficient patients

To gain greater granularity regarding the nature of the circulating TCRαβ^+^CD8^−^ T cells in CD4-deficient patients, we performed single-cell RNA sequencing (scRNAseq) on memory TCRαβ^+^CD8^−^ T cells isolated from healthy donors and patients (P1–P5) that were then stimulated with anti-CD2/CD3/CD28 beads for 20 h. As a comparison, we used TCRαβ^+^CD4^−^CD8^−^ DN T cells from healthy donors, FAS-deficient, and *STAT3* GOF patients. First, we showed that memory TCRαβ^+^CD8^−^ T cells from healthy donors and CD4-deficient, FAS-deficient and *STAT3* GOF patients expressed *CD3G* but not *CD8A* (Fig. S3 G, left and right panels), confirming these cells as being CD8^−^. Moreover, consistent with qPCR data (Fig. 2 A, probe 2), expression of *CD4* mRNA was detected at low levels in patients with mutations leading to premature stop codons (P1–P3) at levels similar to healthy donors in patients with missense variants (P4 and P5) (Fig. S3 G, middle panel). Following a shared nearest neighbor graph-based approach, two main clusters were identified as memory CD4^+^ T cells either in a resting or activated state (Fig. S3 H). As a control, we analyzed five publicly available 10x datasets and found that >99% of unstimulated memory CD4^+^ T cells belonged to the resting cluster, which validated the clustering (Fig. S3 H). Activated cells from healthy donors and patients expressed significantly higher levels of interleukin (*IL)-2*, *IFNG*, *CCL3/4*, *CSF2*, *GZMB*, *ICOS*, and *TNF* than resting cells (Fig. S3 I). The relative abundance of the two populations (resting and activated) was comparable between healthy donors and patients in each batch, despite a difference in stimulation efficacy between the two batches analyzed (resting cells in batch 1: ∼10%; in batch 2: ∼30%) (Fig. S3 J). Furthermore, a pseudobulk PCA of the two populations showed that DN cells from two healthy donors, a *STAT3* GOF patient and a FAS-deficient patient, were transcriptionally distinct from TCRαβ^+^CD8^−^ T cells isolated from healthy donors and patients (P1–P5) (Fig. 4 B). This observation provides further evidence that TCRαβ^+^CD8^−^ T cells in the patients are unrelated to pathogenic DN T cells frequently expanded in monogenic autoimmune conditions such as FAS deficiency.

In both the resting or activated populations, few genes (<30) were found to be significantly differentially expressed between healthy donors and patients. Interestingly, *IL-17F* was downregulated in resting cells of P1–P3 compared with healthy donors (Fig. 4 C, top panel). Moreover, compared with healthy donors, *TIGIT* was upregulated in resting cells from P1–P3 (Fig. 4 C, upper panel) as well as in activated cells from all patients (P1–P5) (Fig. 4 C, bottom panel). In this non-supervised hierarchical clustering, patients with variants in *CD4* that introduced premature stop codons (P1–P3) clustered separately from patients with missense variants (P4 and P5) (Fig. 4 C). Gene set enrichment analysis (GSEA) between TCRαβ^+^CD8^−^ T cells from healthy donors and patients showed reduced expression of genes involved in IL-2/STAT5 signaling in resting (Fig. 4, D and E, upper panel) and/or activated populations such as ICOS (Fig. 4, D and E, bottom panel). Overall, TCRαβ^+^CD8^−^ T cells from CD4-deficient patients transcriptionally resemble TCRαβ^+^CD8^−^ T cells in healthy donors and are distinct from pathogenic DN cells typically expanded in FAS-deficient and *STAT3* GOF patients. Nevertheless, TCRαβ^+^CD8^−^ T cells from CD4-deficient patients exhibit subtle but significant transcriptional differences including reduced expression of some cytokines and responsiveness to IL-2. This likely contributes to mildly impaired CD4 T cell immunity, consistent with the relatively mild infectious phenotypes of these patients.

### Pathogenic *CD4* variants do not affect development or differentiation of NK or myeloid cells

**Figure 5.**
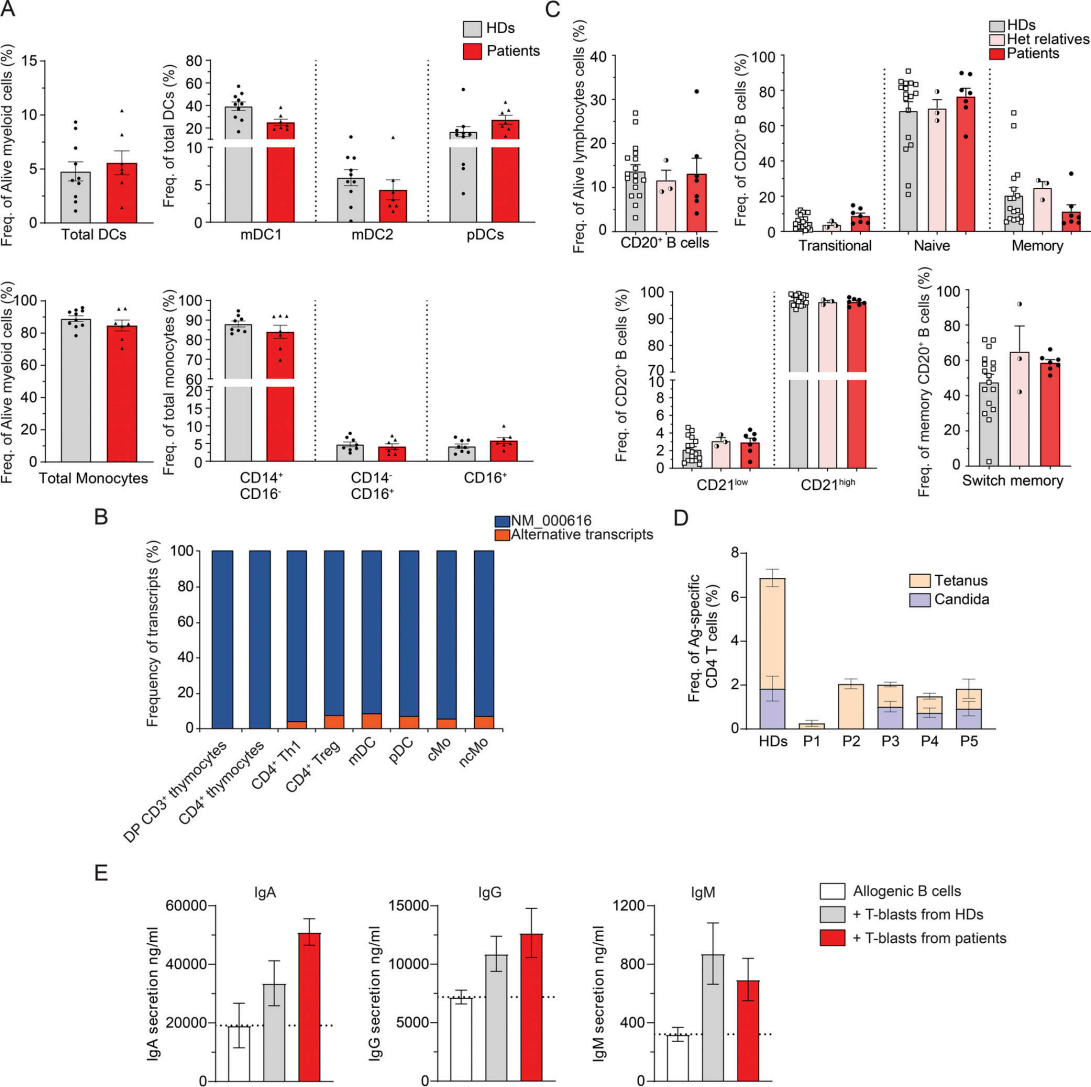
**Impact of CD4 deficiency on leukocyte development and differentiation. (A)** Myeloid cell population frequencies in healthy donors (HDs, *n* = 10) and patients (P1–P7). Total DCs: Lin^−^HLADR^+^CD14^−^CD16^−^; mDC1: CD11c^+^CD123^−^CD141^−^CD1C^+^; mDC2: CD11c^+^CD123^−^CD141^+^CD1C^−^; pDC:CD11c^−^CD123^+^; total Mo: Lin^−^HLADR^+^CD14^+/−^CD16^+/−^. **(B)** Proportion of transcripts expression for different *CD4* expressing cell types as determined by bulk RNA-seq considering sequenced reads spliced from exon 1 with exon 2 (NM_000616), versus with other exons, corresponding to alternative transcripts. **(C)** CD20^+^ B cell population frequencies in HDs (*n* = 18), heterozygous relatives (*n* = 3), and patients (P1–P7). Transitional: CD10^+^CD27^−^; naive: CD10^−^CD27^−^: memory: CD10^−^CD27^+^; isotype switched memory: IgD^−^IgM^−^. **(D)** Frequency of antigen (Ag)-specific CD4 T cells measured by flow cytometry following healthy donors (HD, *n* = 5) or patients’ (P1–P5) PBMCs in vitro stimulation with tetanus toxoid (tetanus, orange), or HKCA (heat-killed *C. albicans*, blue) for 2 days. **(E)** IgA (left), IgG (middle), and IgM (right) secretion detected by ELISA in the supernatant of allogenic B cells cultivated in vitro for 7 days alone (white) or with T-blasts from either healthy donors (gray, *n* = 8) or from patients (red, P1–P7). Data are representative of at least two independent experiments.

**Figure S4.**
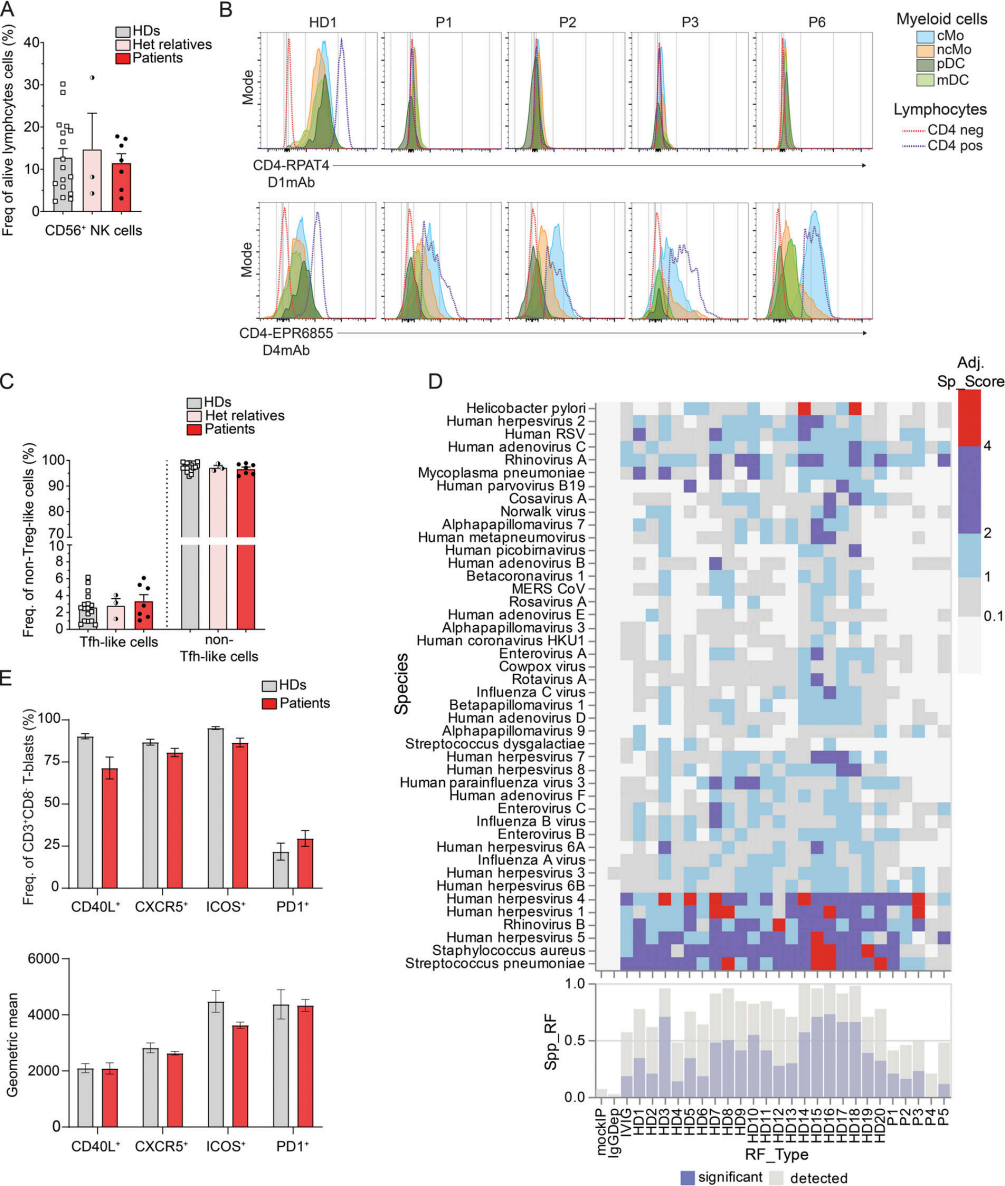
**Effect of CD4 deficiency on NK cells, myeloid cells, Tfh cells, and antiviral antibody responses. (A)** CD56^+^ subset NK cell frequencies in healthy donors (HDs), heterozygous relatives, and patients (P1–P7). **(B)** Flow cytometry following extracellular staining of T-blasts from one representative HD. and patients shown to express isoform 2 (P1–P3, P6). Top: CD4 D1mAb (RPAT4); bottom: CD4 D4mAb (EPR6855). cMo: Lin^−^HLADR^+^CD14^+^CD16^−^, light blue; ncMo: Lin^−^HLADR^+^CD14^+/−^CD16^+^, orange; mDC: Lin^−^HLADR^+^CD14^−^CD16^−^CD11c^+^CD123^−^, light green; pDC: Lin^−^HLADR^+^CD14^−^CD16^−^CD11c^−^CD123^+^, dark green. **(C)** CD3^+^TCRαβ^+^CD8^−^CXCR5^+^ Tfh-like cell frequency in HDs, heterozygous relatives, and patients (P1–P7). **(D)** Antiviral antibody responses to species for which at least one sample tested seropositive by Phage ImmunoPrecipitation Sequencing. “IVIG” corresponds to the mean response for samples from pooled patients on IVIGs, mock IP samples, and IgG-depleted serum. The heatmap shows adjusted virus score values for each sample as a color gradient from blue if antibodies were detected but below our significance cut-off values, through purple to red if the adjusted virus score values were above our significance cut-off values. The bar plot (bottom) illustrates the size of the antibody repertoire for a given sample, indicating the precise number of different species for which peptides were enriched (light blue) and the number of different species for which the adjusted virus score values exceeded the cut-off values for significance (dark blue). **(E)** Top: Frequency of CD3^+^CD8^−^ T-blasts from healthy donors (*n* = 8, gray) and patients (P1–P7, red) expressing canonical Tfh markers CD40L, CXCR5, ICOS, or PD1. Bottom: Geometric mean of CD40L, CXCR5, ICOS, or PD1. Data are representative of at least two independent experiments.

To investigate the impact of *CD4* variants on human leukocytes and generation of specific effector subsets, we assessed the phenotype of NK cells and myeloid cells in patients’ PBMCs (P1–P7) by flow cytometry. No significant differences were found for frequencies of total dendritic cells (DCs, Lin^−^HLADR^+^CD14^−^CD16^−^); nor subsets of DCs (myeloid [m]DC1: CD11c^+^CD123^−^CD141^−^CD1C^+^; mDC2: CD11c^+^CD123^−^CD141^+^CD1C^−^; plasmacytoid [p]DC: CD11c^−^CD123^+^), total monocytes (Mo: Lin^−^HLADR^+^CD14^+/−^CD16^+/−^), or Mo (classical [cMo]: CD14^+^CD16^−^; non-classical [ncMo]: CD14^−^CD16^+^,CD14^+^CD16^+^) (Fig. 5 A); nor NK cells (CD3^−^CD20^−^CD56^+^) (Fig. S4 A) detected in patients (P1–P7) compared with healthy donors. Interestingly, in public datasets (see Materials and methods), expression of alternative *CD4* transcripts (defined by exon 2 and 3 skipping) was found in myeloid subpopulations from healthy donors (mDC: 8.5%; pDC: 6.9%; cMo: 5.7%; ncMo: 7.1%) (Fig. 5 B). However, flow cytometry analysis in PBMCs from healthy donors showed that only expression of CD4 per se could be detected at the surface of myeloid cell subsets (cMo, ncMo, pDC, and mDC) with a lower intensity than CD4 detected in total lymphocytes with both CD4 D3mAb or D4mAb (Fig. S4 B, HD). Nonetheless, for patients expressing isoform 2 WT (P1–P3, P6), surface expression was detected in cMo and ncMo with a similar (cMo) or lower (ncMo) intensity than protein expression in total lymphocytes with CD4 D4mAb (but not with CD4 D3mAb) (Fig. S4 B, bottom panels, P1–P3, P6). Compared with cMo, isoform 2 expression in patients’ DC subpopulations was found to be low (mDC) or not detected (pDC) (Fig. S4 B, bottom panels, P1–P3, P6). Overall, NK and myeloid cell subsets in patients with biallelic *CD4* variants were similar to healthy donors, and isoform 2 expression was detected mostly in patients’ cMo.

### T follicular helper (Tfh) and B cell immunophenotype in CD4-deficient patients

To characterize further the impact of CD4 deficiency on human leukocytes, we analyzed B cells and TCRαβ^+^CD8^−^ T cells from patients and healthy donors’ PBMCs. Frequencies of circulating Tfh-like (CD3^+^TCRαβ^+^CD8^−^CD45RA^−^CXCR5^+^) cells (Fig. S4 C), total B cells (CD3^−^CD20^^+^^) as well as of transitional (CD10^+^CD27^−^), naive (CD10^−^CD27^−^), total memory (CD10^−^CD27^+^), and Ig isotype switched memory (CD27^+^IgD^−^IgM^−^) B cell subsets (Fig. 5 C) were comparable in all patients and healthy donors. Consistent with this, serum levels of total IgM, IgG, and IgA in patients were within reference ranges of healthy donors (Table S3). No serum autoantibodies were detected in any of the patients (Table 1), consistent with a lack of history of clinical autoimmunity.

We performed VirScan (Fig. S4 D) to obtain a general overview of the virome encountered by the patients and measured serum IgG levels specific for known vaccines and pathogens (Table 2). P1–P3 and P5 had been exposed to 5–10 common viruses without severe clinical consequences. The low reactivity of serum detected by VirScan from P4 against the screened viruses is probably explained by reduced exposure due to her young age. To gain resolution on infectious susceptibility to HPV subtypes in the context of CD4 deficiency, we performed a Luminex assay detecting antibodies against 38 different HPV L1 virus-like particles in plasma from P2–P5 and P7 (Table S4). In Kindred B, P2 was seropositive for HPV-1 and HPV-8, and P3 for HPV-17 and HPV-21. In Kindred C, P4 was seropositive for HPV-2, HPV-6, HPV-9, HPV-11, HPV-16, HPV-17, HPV-21, HPV-23, HPV-27b, HPV-36, HPV-41, HPV-75, and HPV-80, whereas P5 was seropositive for HPV-9, HPV-10, HPV-38, and HPV-101. In Kindred E, P7 was seropositive for HPV-15, HPV-23, and HPV-48. These data suggest that CD4-deficient patients had been exposed to different HPV subtypes and developed protective antibody responses. However, P3 developed multiple non-pruritic verrucous skin lesions (HPV3^+^) and genital warts (HPV31^+^) but was seronegative for these HPV subtypes, which suggests a defective specific antibody response.

**Table 2.** Serological data for patients with AR CD4 deficiency

Patient	Mutation	HSV-1 (>1.1)	HSV-2 (>1.1)	CMV (>14)	EBV (>20)	VZV (>135)	Measles (>16.5)	Mumps (>11)	Rubella (>10)	B19 (>1.1)	Anti-HBs Ab (>10)	HCV (>1)	HAV (>1)	HIV (>1)	Others	Age at sampling (years)
P1	c.491delA/491delA	Neg	Neg	155	100 (VCA)	Neg	18	51	Neg	Neg	47	Neg	Neg	Neg	−	13
P2	p.S82*/S82*	34	Neg	116	Neg	1960	ND	ND	ND	3	Neg	Neg	Neg	Neg	−	52
P3	p.S82*/S82*	44	Neg	Neg	Neg	1070	274	36	137	Neg	Neg	Neg	9	Neg	−	50
P4	p.R79C/R79C	1.4	Neg	>180	152	Neg	>300	133	185	Neg	473	Neg	Neg	Neg	−	5
P5	p.R79C/p. R79C	46	Neg	175	55	1740	Neg	Neg	28	28	Neg	Neg	Neg	Neg	−	22
P6	c.1A>G/c.1A>G	ND	ND	ND	ND	Pos	Pos	Neg	Pos	ND	Neg	ND	Neg	Neg	−	23
P7	c.1157-1 G>A/c.1157-1 G>A	ND	ND	>250	>192 (VCA)	ND	ND	ND	ND	ND	ND	ND	ND	Neg	−	45

Ag, antigens; B19, parvovirus B19; CMV, cytomegalovirus; DT, doubtful result; EBV, Epstein–Barr virus; HAV, hepatitis A virus; HBs, hepatitis B surface Ag; HSV, herpes simplex virus; ND, not determined; VZV, varicella-zoster virus; −, uninfected with other known pathogens.

### T and B cell function in the context of CD4 deficiency

To further explore the function of T cells detected in patients with biallelic *CD4* variants, we first quantified antigen-specific T cell responses. PBMCs from healthy donors and patients P1–P5 were challenged in vitro with tetanus toxoid, as an example of a common vaccine, or heat-killed *Candida albicans* (HKCA) as an example of a commensal pathogen. After 2 days, the frequency of antigen-specific responder cells (CD3^+^CD8^−^OX40^+^CD25^+^) was measured by flow cytometry (Zaunders et al., 2009) We detected tetanus-specific and *Candida*-specific CD3^+^CD4^−^CD8^−^ cells in 3/5 (mean: 0.9%) and 5/5 (mean: 1.15%) CD4-deficient patients, respectively (Fig. 5 D). While the magnitude of responses detected in the CD4-deficient patients was above background, they were generally reduced two- to threefold compared with healthy donors (*n* = 9; 1.85% tetanus- and 4.5% *Candida*-specific CD4^+^ T cells) (Fig. 5 D). Thus, these data established that CD4-deficient individuals can generate functional antigen-specific T cell responses; however, the magnitude of these responses tends to be lower than those in healthy donors.

Upon interactions with B cells, Tfh cells play an essential role in the adaptative immune response by enhancing B cell survival, proliferation, differentiation, and antibody secretion (Tangye and Ma, 2021; Tangye et al., 2013). Thus, to characterize the impact of CD4 deficiency on the capacity of CD3^+^CD8^−^ T cells to provide help to B cells, we used an in vitro culture system that we previously developed to investigate Tfh function of CD4^+^ T cells isolated from healthy donors and patients with other defined IEI (Ma et al., 2005, 2009, 2015). Polyclonal CD3^+^TCRαβ^+^ T-blasts generated from healthy donors and CD4-deficient patients were cocultured with allogeneic B cells for 7 days. Ig secretion was then measured as a readout of T cell–dependent B cell differentiation. We observed an increase of IgA (2.7-fold), IgG (1.5-fold), and IgM (2-fold) secreted by allogeneic B cells in the presence of cocultured T-blasts from healthy donors compared with B cells cultured alone (Fig. 5 E). Importantly, a similar level of augmentation of IgA, IgG, and IgM secretion was achieved when B cells were cocultured with patient-derived CD8^−^ T-blasts (Fig. 5 E). Consistent with these findings, expression of canonical Tfh markers including ICOS, CXCR5, CD40L, and PD-1 on T-blasts from CD4-deficient patients was comparable with that observed for healthy donors (Fig. S4 E). Overall, these results demonstrate that T cells from CD4-deficient patients have a preserved capacity to provide help to B cells in vitro.

### T helper (T_H_) cell cytokine production and secretion in human CD4 deficiency

**Figure S5.**
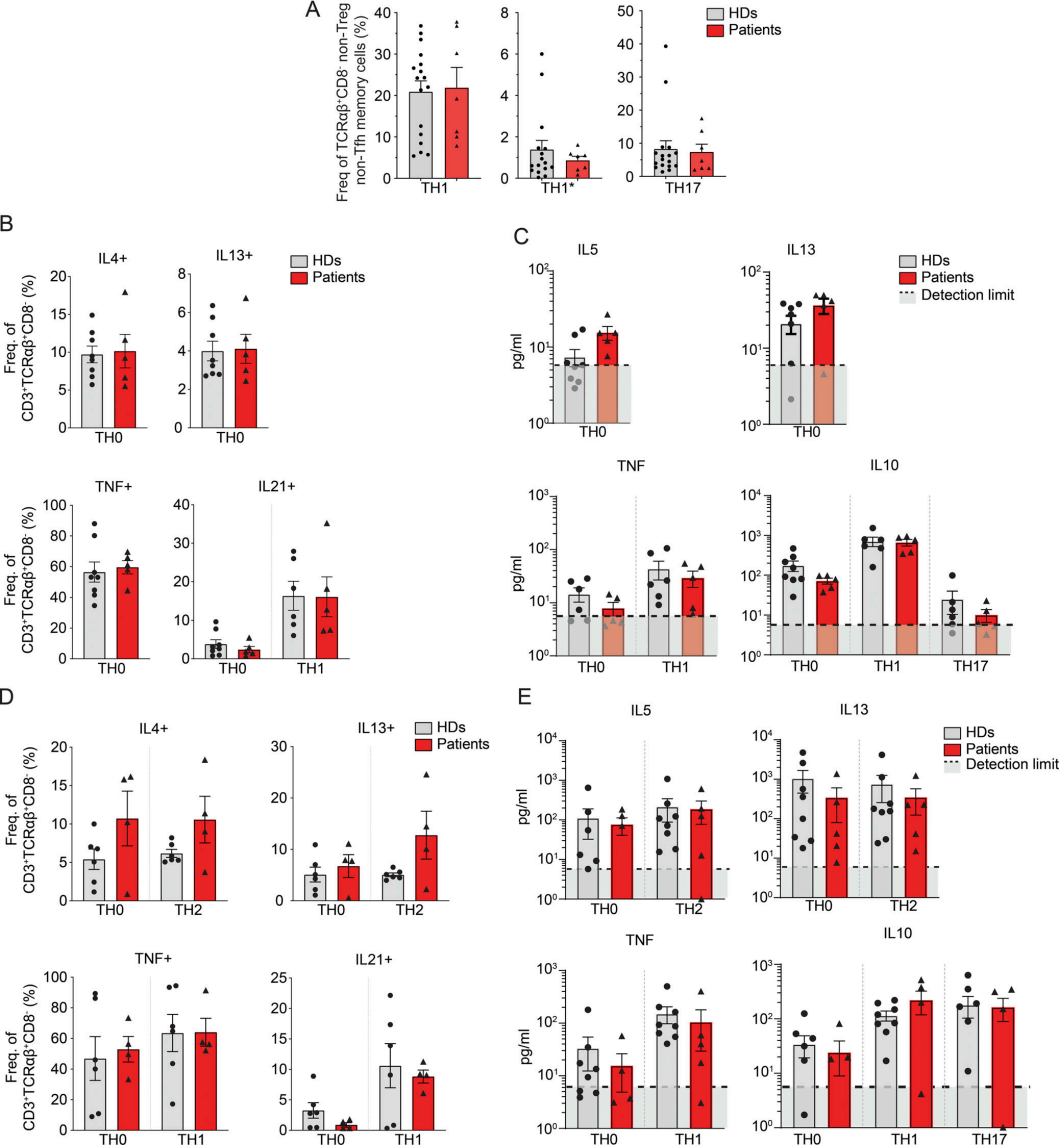
**In vitro and ex vivo characterization of lymphocytes subsets in CD4-deficient patients. (A–E)** Frequency of indicated subset of healthy donors (HD) and patients assessed by flow cytometry. **(A)** CD3^+^TCRαβ^+^CD8^−^CXCR5^−^CD45RA^−^CXCR5^−^ gated cells. Th1: CXCR3^+^CCR6^−^; Th1*: CXCR3^+^CCR6^+^; Th17: CXCR3^−^CCR6^+^. **(B)** Polarized memory CD3^+^TCRαβ^+^CD8^−^ cell intracellular cytokine production. **(C)** Polarized memory CD3^+^TCRαβ^+^CD8^−^ cell intracellular cytokine secretion. The limit of detection is indicated by a dashed line and a gray area. **(D)** Polarized naive CD3^+^TCRαβ^+^CD8^−^ cell intracellular cytokine production. **(E)** Polarized naive CD3^+^TCRαβ^+^CD8^−^ cell intracellular cytokine secretion. The limit of detection is indicated by a dashed line and a gray area. Data are representative of at least two independent experiments.

Additional flow cytometric analyses were performed on TCRαβ^+^CD8^−^ T cells from patients and healthy donor PBMCs. Proportions of memory TCRαβ^+^CD8^−^ subpopulations (T_H_1: CXCR3^+^CCR6^−^; T_H_1*: CXCR3^+^CCR6^+^; T_H_17: CXCR3^−^CCR6^+^) were within normal ranges in the patients (P3–P7), while T_H_1 cells were increased in P1 and P2 (Fig. S5 A).

**Figure 6.**
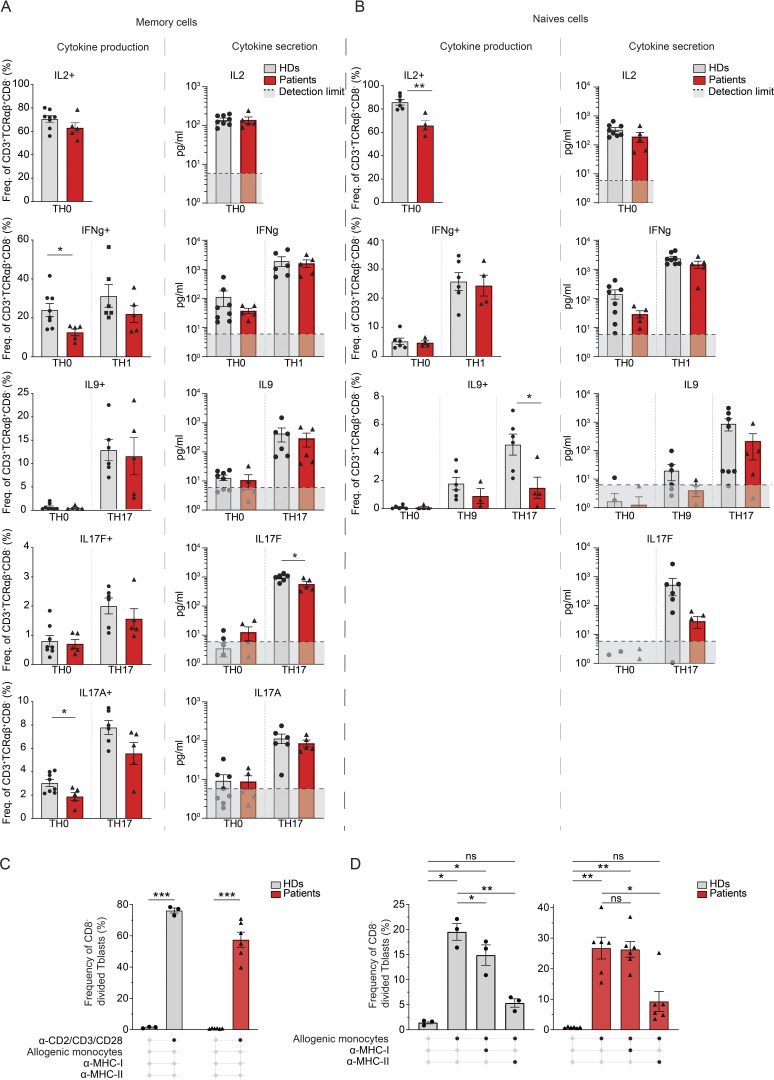
**Effector function of CD3**^**+**^**TCRαβ**^**+**^**CD8**^**−**^ **T cells due to CD4 deficiency. (A and B)** Frequency of indicated subset of healthy donors (HDs) and patients assessed by flow cytometry. Statistical analysis by unpaired Student’s *t* test. *P < 0.05; **P < 0.01. **(A)** Polarized memory CD3^+^TCRαβ^+^CD8^−^ cells intracellular cytokine production (left) and secretion (right). The cytokine secretion limit of detection is indicated by a dashed line and a gray area. **(B)** Polarized naive CD3^+^TCRαβ^+^CD8^−^ cells intracellular cytokine production (left) and secretion (right). The cytokine secretion limit of detection is indicated by a dashed line and a gray area. **(C and D)** Stimulation conditions are represented below histograms. Statistical analysis by one-way ANOVA with multiple comparisons (Tukey). ns: non-significant; *P < 0.05; **P < 0.01; ***P < 0.001. (C) Frequency of CD8^−^ divided T-blasts from three healthy donors and six patients (P1–P6) after 5 days either non-stimulated or incubated with anti-CD2/CD3/CD28 beads or (D) either non-stimulated or cocultured with healthy donor allogenic pan Mo (mixed lymphocyte reaction) in presence or absence of HLA class I or class II blocking antibodies. Data are representative of at least two independent experiments.

We next assessed the consequences of CD4 deficiency on TCRαβ^+^CD8^−^ T_H_ cell function both in vitro (naive cells) and ex vivo (memory cells). TCRαβ^+^CD8^−^ T cells were cultured with anti-CD2/CD3/CD28 mAb-coated beads alone (T_H_0) or under T_H_1 (+IL-12) or T_H_17 (+TGFβ, IL-1, IL-6, IL-21, IL-23) polarizing conditions. The capacity of TCRαβ^+^CD8^−^ memory T cells from CD4-deficient patients (P1–P5) to produce and secrete effector cytokines IL-4, IL-5, IL-13, IL-10, and TNFα under T_H_0 conditions and specific polarizing condition was similar to corresponding cells from healthy donors (Fig. S5, B and C). However, compared with healthy donors, we observed significant decreases in IFN-γ and IL-17A production under T_H_0 polarizing conditions by memory TCRαβ^+^CD8^−^ T cells from patients with *CD4* mutations (Fig. 6 A). Reduced production of IFN-γ and IL-17 were also observed under T_H_1 and T_H_17 polarizing conditions, respectively. Indeed, consistent with scRNAseq data (Fig. 4 C), patients’ memory cells secreted significantly less IL-17F following culture under T_H_17 condition compared with healthy donors (Fig. 6 A).

To determine whether these defects in cytokine production by CD4-deficient memory cells were cell-intrinsic or secondary to defects during in vivo priming, we next assessed the capacity of naive TCRαβ^+^CD8^−^ T cells to differentiate into cytokine-producing cells in vitro. Similar to responses of memory TCRαβ^+^CD8^−^ T cells, the global ability of naive TCRαβ^+^CD8^−^ T cells to produce and secrete cytokines was generally comparable with healthy donors (Fig. 6 B; and Fig. S5, D and E). However, production of IL-2 and IL-9 by patients’ naive TCRαβ^+^CD8^−^ T cells was significantly decreased compared with corresponding cells from healthy donors (Fig. 6 B). Similar trends were observed for IL-17F secretion (Fig. 6 B). Overall, these data revealed that CD4 deficiency results in a modest cell-intrinsic impairment in the ability of naive TCRαβ^+^CD8^−^ T cells to differentiate into T_H_1-type, T_H_17-type, and IL-9^+^ effector cells.

### HLA class II restriction and alloreactivity

HLA class II–restricted antigen presentation is crucial for CD4^+^ T cell development, selection, and function (Nekrep et al., 2003). Therefore, we investigated the capacity of patients’ T cells to proliferate in vitro upon allo-stimulation, as well as the dependence on HLA class II, by mixed lymphocyte reactions. T-blasts from patients (P1–P6) and healthy donors were labeled with division-tracking dyes and cocultured with Mo isolated from third-party healthy allogeneic donors in the absence or presence of blocking antibodies against HLA class I or class II or incubated with anti-CD2/CD3/CD28 beads to induce maximal proliferation.

Compared with non-stimulated conditions, we observed a highly significant increase in the frequency of divided CD8^−^ T-blasts from healthy donors (mean: 74.7%, range [73–78.4%]) and patients (mean: 57.5%, range [39.8–70.9%]) following 5 days of stimulation with anti-CD2/CD3/CD28 beads (Fig. 6 C). Importantly, we also detected significant induction of proliferation by responder CD8^−^ T cells from both healthy donors (mean: 19.5%, range [16.4–22.1%], Fig. 6 D, left) and patients (mean: 26.7%, range [15.5–40.2%], Fig. 6 D, right) upon coculture with allogeneic Mo.

Blocking HLA class I had modest or no effect on Mo-dependent proliferation of responder T cells from healthy donors (mean: 14.9%, range [10.9–17.6%], Fig. 6 D, left) or patients (mean: 26.3%, range [18–37%], Fig. 6 D, right). Strikingly, HLA class II blocking mAbs inhibited proliferation induced by allogeneic Mo of CD8^−^ T-blasts from healthy donors (mean: 5.3%, range [3.7–6.4%], Fig. 6 D, left) and patients (mean: 9.8%, range [3.5–25.2%], Fig. 6 D, right) by >60–70%. Altogether, these results demonstrate that T-blasts from CD4-deficient patients can respond as robustly as CD4-sufficient T-blasts from healthy donors to HLA class II–restricted antigens presented by allogeneic Mo.

## Discussion

When expressed at the surface of T cells, a key function of the CD4 co-receptor is to enhance TCR activation by enabling signal transduction via LCK following recruitment to the TCR-CD3-HLA class II macrocomplex (Glassman et al., 2018; Horkova et al., 2023). The canonical isoform encoded by *CD4* (CD4 per se) interacts directly with HLA class II via its distal ectodomains D1 and D2 (Clayton et al., 1989; Fleury et al., 1991; Glassman et al., 2018). Furthermore, reciprocal binding can occur between the membrane-proximal ectodomains (D3 and D4) of CD4 and CD3 subunits (Glassman et al., 2018; Lynch et al., 1999; Nakamura et al., 2003; Vignali et al., 1996; Wu et al., 1997) as well as through intracellular interactions involving recruited LCK (Li et al., 2017; Mingueneau et al., 2008; Xu and Littman, 1993). Recent studies in humans identified patients with partial or complete LCK deficiency. Interestingly, CD4 expression was decreased in T cells from LCK deficient patients, suggesting that recruited LCK plays a crucial role in stabilizing CD4 on the surface of T cells (Keller et al., 2023; Lanz et al., 2023; Lui et al., 2024). CD4-mediated interactions with CD3 increase the duration of cognate TCR–HLA class II interplay and therefore ensure scalable signaling which determines the duration and magnitude of responses of CD4^+^ T cells (Glassman et al., 2018).

In this study, we first demonstrated that T cells from healthy donors naturally express not only the well-characterized predominant *CD4* transcript encoding the CD4 protein per se but also a shorter protein (isoform 2) comprising extracellular D4, transmembrane, and intracellular domains, which is expressed at the surface of TCRαβ^+^CD8^−^ T cells. From a structural perspective, the lack of D1 and D2 domains prevents isoform 2 from directly binding HLA class II (Clayton et al., 1989; Fleury et al., 1991). Second, we identified five novel patients with an infectious phenotype carrying homozygous *CD4* variants leading to CD4 deficiency. While there was significant overlap in clinical features of all CD4-deficient patients, some variability was detected; this may reflect somatic, germline, or epigenetic regulators, environmental factors, patient age, and/or pathogen exposure. Third, we performed in-depth analyses of all known (*n* = 7) CD4-deficient individuals. Our studies consistently revealed that patients’ PBMCs expressed detectable amounts of the alternative naturally occurring transcript (with a start site located in exon 6 of *CD4*) encoding the shorter isoform 2. Indeed, we detected surface expression of WT isoform 2 on TCRαβ^+^CD8^−^ T cells from all patients with variants causing premature stop codons (P1–P3) or start loss (P6) in *CD4*. Moreover, we observed that the missense variant identified in P4 and P5 (R79C) did not prevent surface CD4 expression but rather affected protein folding due to the introduction of a cysteine at position 79 (no detection with D1 or D3 mAbs). Despite an apparent inability to associate with HLA class II, our data suggest that mutant CD4 (R79C) as well as WT isoform 2 detected in patients P1–P6 can interact with LCK and enhance ZAP70 phosphorylation and downstream TCR signaling in vitro; it remains to be determined whether this also occurs in vivo. In P7, surface expression of CD4 could be detected (D4mAb) but the mutated protein lacked catalytic function (no interaction with LCK nor ZAP70 phosphorylation enhancement). However, all TCRαβ^+^CD8^−^ T cells from the patients had intact TCR signaling upon CD3 engagement, as seen for the other CD4-deficient patients. The similarity of immunological and clinical phenotypes between patients expressing mutant CD4 (P4 and P5), WT isoform 2 (P1–P3 and P6), mutant CD4 and mutant isoform (P7) indicates that all patients have CD4 deficiency and highlight the diversity of compensatory mechanism in T cell development and function.

Detailed immunophenotyping of patient PBMCs revealed normal proportions of TCRαβ^+^ and TCRγδ^+^ T cells. However, all patients exhibited a complete absence of CD4-expressing T cells as detected using routine mAbs to detect CD4, increased frequencies of CD8^+^ T cells, and an expansion of TCRαβ^+^CD4^−^CD8^−^ T cells. In mice, targeted deletion of *Cd4* did not affect numbers of T and B cells in lymph nodes nor numbers of thymic CD8^+^ T cells, although CD8^+^ T cells were increased in the periphery. Expression of CD3 and TCRαβ on *Cd4*-deficient lymphocytes was also intact (Rahemtulla et al., 1991). However, a higher number of TCRαβ^+^ DN T cells was present in the thymus and periphery of *Cd4*^−/−^ mice compared with *Cd4*^*+/+*^ mice (Kondo et al., 1996; Pearce et al., 2004; Rahemtulla et al., 1993; Sha and Compans, 2000). In humans, conventional CD4^−^CD8^−^ DN T cells are present at low frequencies in healthy donors. However these cells are dramatically expanded in individuals with some immune dysregulatory conditions, such as ALPS due to genetic variants affecting the FAS/FASL pathway (Bleesing et al., 2001; Dowdell et al., 2010; Fisher et al., 1995; Maccari et al., 2021; Magerus-Chatinet et al., 2009; Völkl et al., 2016), or in patients with heterozygous *STAT3* GOF (Flanagan et al., 2014; Haapaniemi et al., 2015; Milner et al., 2015; Nabhani et al., 2017) or homozygous *PDCD1* (PD-1) (Ogishi et al., 2021) variants. It was thus possible that the increase in TCRαβ^+^ DN T cells in patients’ PBMCs resulted from a bona fide expansion of pathogenic DN T cells. However, in CD4-deficient patients, our data indicates this is highly unlikely for the following reasons: First, detailed TCRαβ sequence analysis demonstrated memory TCRαβ^+^CD4^−^CD8^−^ T cells from the patients were comparable with memory TCRαβ^+^CD4^+/−^CD8^−^ T cells from healthy donors and were predicted to correspond to CD4^+^ T cells. Second, transcriptomic studies showed patients’ memory TCRαβ^+^CD8^−^ T cells to be mostly comparable with healthy donor cells but distinct from pathogenic TCRαβ^+^ DN T cells expanded in FAS-deficient patients. Third, >95% of TCRαβ^+^ DN cells in CD4-deficient patients could be defined as conventional DN cells (CD38^−^CD45^+/−^), as observed for healthy donors, rather than pathogenic DN (CD38^+^CD45^+/−^) cells that are enriched (50%) in FAS deficiency. Thus, we conclude that the TCRαβ^+^CD8^−^CD4^−^ T cells that are markedly increased in CD4-deficient patients correspond to T cells that are ordinarily selected to and comprise the CD4^+^ T cell lineage in healthy individuals.

HLA class II–restricted naive CD4^+^ T cells are selected by a very stringent and complex process in the thymus where the avidity and duration of MHC-TCR interactions are fundamental (Glassman et al., 2018; La Gruta et al., 2018; Singer, 2002; Van Laethem et al., 2022; Zareie et al., 2020). Remarkably, Singer and colleagues recently reported a new FlipFlop mouse model where the *Cd4* and *Cd8α* gene loci encode the opposite co-receptor protein (Shinzawa et al., 2022). This study showed that T cell lineage fate was determined by cis-regulatory elements in co-receptor loci which impact CD4 or CD8 protein expression and TCR signaling duration, regardless of the co-receptor protein encoded (Shinzawa et al., 2022). However, this study also showed that in vivo protective immunity strictly requires the *Cd4* loci to encode an MHC class II–specific co-receptor protein and similarly for *Cd8*/MHC class I (Shinzawa et al., 2022). Another interesting study in mice recently showed that the CD4-LCK interaction was not required for commitment of HLA class II–restricted thymocytes to the CD4^+^ T cell lineage (Horkova et al., 2023). Rather, this study suggested that CD4 (coupled with LCK) is crucial for acquisition of effector function of peripheral mature T cells (Horkova et al., 2023). Moreover, functional studies of the expanded TCRαβ^+^ DN T cells in *Cd4*^−/−^ mice revealed they were HLA class II–restricted and appeared to have effector attributes characteristic of CD4^+^ T cells rather than CD8^+^ T cells (Pearce et al., 2004; Rahemtulla et al., 1993). TCRαβ^+^ DN T cells from *Cd4*^−/−^ mice challenged with different pathogens produced lower levels of cytokines such as IL-2, IL-4, IL-12p40, IL-15, TNF-α, or IFN-γ compared with *Cd4*^+/+^ T cells (Bitsaktsis et al., 2004; Kawakami et al., 2001; Kondo et al., 1996; Mohammed et al., 2000; Niethammer et al., 2001; Pearce et al., 2004; Serbina et al., 2001).

Consistent with these studies in mice, we found that a population of TCRαβ^+^ DN T cells that are quantitatively and qualitatively similar to canonical CD4^+^ T cells in healthy donors can be generated in all CD4-deficient patients. Indeed, patients’ TCRαβ^+^ DN T cells exhibited HLA class II–restricted proliferation upon coculture with allogenic Mo. Moreover, serological and cellular analyses showed that CD4-deficient patients can generate functional antigen-specific T cell and B-cell/Ab responses, consistent with preserved capacity of patient CD8^−^ T cells to provide help to induce B cell differentiation in vitro. Furthermore, T_H_ subpopulation frequencies were largely intact in patients, based on surface phenotypes, and there were only subtle functional defects compared to CD4^+^ T cells from healthy donors. Specifically, patients CD3^+^CD8^−^ T cells exhibited reduced IL-2 signaling as well as mild cell-intrinsic impairments in differentiating into T_H_1-type, T_H_17-type, and IL-9^+^ effector cells. Human CD4^+^ T cells that commit to a T_H_1 or T_H_17 fate or that produce IL-9 have important roles in host defense against different infections, particularly those caused by fungal and staphylococcal species (Badolati et al., 2023; Bröker et al., 2016; Brown et al., 2015; McDonald, 2012; Puel et al., 2012; van de Veerdonk and Netea, 2010; Zielinski et al., 2012). While these cells have not been directly implicated in protection against HPV, our findings indicate that further functional analyses of the roles of specialized subsets of human CD4^+^ T cells in protective responses are warranted.

While each of the patients with inherited CD4 deficiency displayed an infectious phenotype, it was, however, milder than that reported for people with CD4^+^ T cell deficiency due to HIV infection, HLA class II deficiency, or idiopathic CD4^+^ T cell lymphopenia (Al-Herz et al., 2013; Ben-Mustapha et al., 2013; Gottlieb et al., 1981; Lisco et al., 2023; Masur et al., 1981; Ouederni et al., 2011; Rozmus et al., 2013; Small et al., 1983; Vieira et al., 1983). P1 presented with multiple infectious episodes including recurrent pneumonia, chronic sinusitis, urinary tract infection, diarrhea, iridocyclitis, and endophthalmitis; P2 with Whipple’s disease, and his brother P3 suffered from tuberculosis and extensive verrucous dermatitis (HPV3^+^ and HPV31^+^); incomplete clinical penetrance was observed in Kindred C as P4 presented with recurrent pulmonary infections and BCG-itis, chronic diarrhea, cryptosporidiosis infection and oral candidiasis while P5 was largely asymptomatic. Interestingly, P5 was found to express normal total *CD4* mRNA level, and higher CD4 protein expression (D4mAb) was detected at the surface of TCRαβ^+^ CD8^−^ T cells compared with P4. P6 presented with recurrent otitis and episodes of upper respiratory infections but also multifocal pneumonia due to rhinovirus and enterovirus and a history of recalcitrant HPV-related warts (Lisco et al., 2021). P7 had a long history of persistent extensive warts in both feet and hands (Fernandes et al., 2019). In contrast, *Cd4*^−/−^ mice showed increased susceptibility to a broad range of experimentally induced pathogen infections, including *Staphylococcus aureus* (Mohammed et al., 2000), lymphocytic choriomeningitis virus (Battegay et al., 1994; Fuller et al., 2004; Khanolkar et al., 2004; Pien et al., 2002; Sun and Bevan, 2004), *Cryptococcus neoformans* (Kawakami et al., 2001), *Mycobacterium tuberculosis* (Serbina et al., 2001), rotavirus (VanCott et al., 2001), prions (Lewicki et al., 2003), *Ehrlichia* (Bitsaktsis et al., 2004), or hepatitis virus (Chakravarty et al., 2020). Overall, these findings highlight the central role of CD4 in adaptive immunity and the importance of T_H_ function for host defense. Nevertheless, it is striking and perhaps surprising that these seven patients are alive (5–61 yo) and relatively well. They have been resistant to a wide range of common viruses, bacteria, fungi, and parasites, including life-threatening pathogens for patients with HLA class II deficiency (Al-Herz et al., 2013; Ben-Mustapha et al., 2013; Ouederni et al., 2011; Rozmus et al., 2013). Additional in-depth studies are now required to further elucidate the exact roles of CD4 in different lymphocyte but also myeloid subsets as well as unravel its contribution to the TCR-CD3-HLA class II complex affinity interplay and the consequences on downstream signaling activation. It will also be important to conduct further studies using *Cd4*^−/−^ mice, which share some similarities with these patients, to investigate the role of potential alternative isoform(s) in phenotypes previously reported in these mouse models.

## Materials and methods

### Healthy donors and patients

P1 is resident in Colombia, P2 is resident in France, P3 and P7 are resident in Portugal, P4 and P5 are resident in Israel, P6 is resident in the United States of America (Table 1). Buffy coats collected from healthy donors were purchased from the Australian Red Cross Blood Service. Written informed consent was obtained from participants or their guardians. Experiments using samples from human subjects were conducted in accordance with local regulations and with the approval of the Sydney Local Health District Human Research Ethics Committee, Royal Prince Alfred Hospital, Camperdown New South Wales Australia (Protocol X16-0210/LNR/16/RPAH/257), and institutional review boards of the Universidad de Antioquia (F8790-07–0010), Rockefeller University, Necker Hospital for Sick Children, Sheba Medical Center Israel, National Institute of Allergy and Infectious Diseases clinical protocols, National Institutes of Health (http://ClinicalTrials.gov identifiers NCT00867269, NCT00001281, NCT00039689, and NCT00001316), and Comité de Ética de la Investigación con medicamentos. del area de Salud de Salamanca.

### Genetics

Patients were genotyped from DNA extracted from whole blood with the Genome-Wide Human SNP Array6.0 and/or WES with SureSelect Human All Exon V6 from Agilent. GoTaq DNA Polymerase (#M3005; Promega) was used with melting temperature (Tm) of 60°C. Amplicons were then sequenced by the Sanger sequencing method with Big Dye Terminator v3.1 (Thermo Fisher Scientific) and subjected to capillary electrophoresis (#A30469; Applied Biosystems 3500xL system; Thermo Fisher Scientific).

### Cell culture and stimulation

HEK293T (#CRL3216; ATCC) were cultured in Dulbecco modified Eagle’s minimal high glucose (#11965118; DMEM; Gibco) supplemented with decomplemented 10% fetal bovine serum (#SH30406.02, FBS; Hyclone). THP1 and D1.1 (#CRL10915; ATCC) were cultured in Roswell Park Memorial Institute (RPMI) 1640 Medium (#11875119; Gibco) supplemented with 10% FBS. T-blasts were cultured in ImmunoCult-XF T cell Exp Medium (#10981; Stemcell) in the presence of IL-2 (#78220.1, 10 ng/ml; Stemcell) and primed every 2 wk with ImmunoCult Human CD3/CD28/CD2 T cell Activator (#10970; Stemcell). All cells were grown at 37°C under an atmosphere containing 5% CO_2_. HEK293T cells were plated at a density of 600,000 cells per well in 6-well plates.

### RT-qPCR, cDNA, and transcripts characterization

RNA was extracted with the Zymo Quick-RNA microprep kit (#R1051; Zymo). Any genomic DNA was removed using Zymo Spin-Away filters (#C10006-250-F). RNA was reversed-transcribed with the High-Capacity RNA-to-cDNA Kit (#4387406; Applied Biosystems) according to the manufacturer’s protocol. qPCR was performed on cDNA with TaqMan Fast Advanced Master Mix (#4444557; Thermo Fisher Scientific) on a QuantStudio 7 Pro Real-Time PCR System (Applied Biosystems) with the following probes: CD4 exons 2–3 (NM_000616.4, #Hs01058404_g1), CD4 exons 6–7 (NM_000616.4, #Hs01058407_m1), and *GUSB* (#4326320E). Full-length *CD4* cDNA amplification was performed on cDNA using the following primers targeting non-coding exon 1 (5′-TGA​GAA​GCA​GCG​GGC​AAG​AA-3′) and 3′UTR (5′-GAT​CCC​ACT​TGC​AGC​CTC​C-3′) with MyTaq DNA polymerase (#BIO-21105; Bioline) and a Tm of 60°C. To identify different *CD4* transcripts, TOPO TA cloning was performed on amplicons with TOPO TA cloning kit (#K450001; Thermo Fisher Scientific) according to the manufacturer’s protocol. Sanger sequencing (as previously described) was performed on bacteria clones using M13R (5′-CAG​GAA​ACA​GCT​ATG​AC-3′) and T7 (5′-TAA​TAC​GAC​TCA​CTA​TAG​G-3′) primers. To quantify different *CD4* transcripts, automated capillary electrophoresis separation (LabChip GX Touch HT Nucleic Acid Analyzer, #CLS137031; PerkinElmer) was performed on amplicons with HT DNA NGS 3K Reagent Kit (#CLS960013; PerkinElmer), followed by a bioinformatic analysis with LabChip GX Software Version 5.4.2227.0 SP2.

### Site-directed mutagenesis and transient transfection

Empty vector and plasmids containing DDK-tagged *CD4* cDNA (NM_000616 and NM_001195016) were obtained from a commercial source (#PS100001, #RC206453, and #RC233384, respectively, Origene). Constructs carrying single-nucleotide mutant alleles were generated from these plasmids by mutagenesis with appropriate primers, with the Q5 Site-Directed Mutagenesis kit (#E0552S; New England Biolabs), according to the manufacturer’s protocol. Plasmids were amplified in competent *E. coli* cells (One Shot TOP10 Chemically Competent, #C404003; Thermo Fisher Scientific). HEK293T cells were transiently transfected with the various constructs at a concentration of 1 µg/ml with the Lipofectamine 3000 transfection kit (#L3000-015; Thermo Fisher Scientific), according to the manufacturer’s protocol.

### Western blotting and immunoprecipitation (IP)

Protein extracts were prepared by mixing cells with total cell lysis buffer (50 mM Tris-HCl, pH 7.4, 150 mM NaCl, 0.5% Triton X-100, and 2 mM EDTA) or IP buffer (150 mM Tris-HCl, pH 8, 150 mM NaCl, 1% Triton X-100, and 5 mM EDTA) supplemented with protease inhibitors (#4693124001; Complete Mini Protease Inhibitor Cocktail; Roche) and phosphatase inhibitor cocktail (#4906837001; PhoStop; Roche). The total cell lysis buffer was also supplemented with 0.1 mM dithiothreitol (Thermo Fisher Scientific). Lysis was performed on ice for 40 min (hard vortex every 10 min). For total cell lysis, equal amounts of protein, according to a Bradford protein assay (#5000006; Bio-Rad), were resolved by SDS-PAGE in AnykD Criterion TGX precast polyacrylamide gel (#5671123; Bio-Rad) and transferred to an Immun-Blot Low Fluorescence PVDF Membrane (#162-0263; Bio-Rad). For IP, isotype clearing was performed overday followed by specific IP overnight with antibodies directed against CD4 domain 4 (unconjugated, #ab133616, clone EPR6855; Abcam) or LCK (unconjugated, #ab229379, clone EPR20798-107; Abcam), and protein A/G magnetic beads (#88803; Pierce) were resolved as total cell lysates. Membranes were probed with antibodies directed against CD4 domain 1 (unconjugated, #ab133313, clone EPR3942; Abcam), CD4 domain 4 (unconjugated, #ab133616, clone EPR6855; Abcam), CD4 domain 4 (HRP-conjugated, #ab195842, clone EPR6855; Abcam), LCK (unconjugated, #ab229379, clone EPR20798-107; Abcam), DDK-tag (unconjugated, #14793, clone D6W5B; Cell Signaling), or GAPDH (unconjugated, #sc-32233, clone 6C5; Santa Cruz). Primary antibodies were detected by incubation with goat anti-rabbit IRDye 680RD (#926-68071; Licor) and donkey anti-mouse IRDye 800CW (#926-32212; Licor). Binding was detected with Odyssey CLx Imager (Licor). The Chameleon Duo Prestained Protein Ladder (#928-60000; Licor) was used to provide a molecular weight marker. Images were analyzed with Image Studio software (Licor).

### Flow cytometry on transfected HEK293T

CD4 expression at the cell surface was assessed on transiently transfected HEK293T by extracellular staining with conjugated monoclonal antibodies directed against either CD4 domain 1 (clone RPAT4, #300514; APC; BioLegend), CD4 domain 3 (clone SK3, #565994; APC; BD Pharmingen), or CD4 domain 4 (AF647, #ab196147, clone EPR6855; Abcam). All cells were also stained with the Zombie UV fixable viability dye kit (#423107; BioLegend). Cells were acquired on a BD FACSymphony A5 Cell Analyzer (BD Bioscience) and analyzed with FlowJo Software. For the gating, doublet and dead cells were excluded.

### Lattice microscopy on electroporated D1.1 cells

Constructs were transiently expressed in Jurkat D1.1 cells through electroporation transfection (MPK1096; Neon Transfection System, Thermo Fisher Scientific). Cells were rinsed twice in PBS and resuspended in Buffer R (1 × 10^6^ cells/40 µl) with 1 µg of plasmid. Three consecutive 10-ms and 1,350-V electrical pulses were discharged through the cell suspension before cells were placed in 20% FBS-RPMI and incubated at 37°C 5% CO_2_ for 18–24 h to allow time for expression.

After transfection, cells were seeded on poly-L-lysine–coated glass coverslips before fixation with 4% PFA for 20 min at ambient temperature. The plated cells were then rinsed twice with PBS and permeabilized at ambient temperature for 10 min with 0.1% Triton X-100. Non-specific blocking was then performed in 5% BSA-PBS for 2 h at ambient temperature before incubation in 4.9 µg/ml primary anti-FLAG solution overnight at 4°C. Cells were then incubated in 0.5 µg/ml secondary anti-mouse Dylight-488 solution for 1 h at ambient temperature before a light fixation for 10 min with 1% PFA. After the second fixation, cells were incubated in 1 µg/ml anti-CD45-Alexa647 for 1 h at ambient temperature before a final rinse with blocking buffer. All antibodies were diluted in 1% BSA-PBS and cells were rinsed twice with 1% BSA-PBS between all labeling steps. Labeled cells were imaged on a commercial 3i Lattice Lightsheet microscope at 37°C in PBS.

### CD4 staining of T-blasts and PBMCs from patients and healthy donors using multiple anti-CD3 mAbs

T-blasts were incubated with conjugated monoclonal antibodies directed against CD3 (#300436, BV570, clone UCHT1; BioLegend), CD4 domain 3 (#612963, BUV661, clone SK3; BD Horizon), CD8 (#612942, BUV496, clone RPAT8; BD Horizon), CD20 (#612905, BUV805, clone 2H7; BD Horizon), TCRαβ (#613014, BUV737, clone T10B9.1A-31; BD Horizon), TCRγδ (#613014, BV711, clone 11F2; BD OptiBuild) and either CD4 domain 1 (clone RPAT4, #300514; APC; BioLegend), CD4 domain 4 (#ab196147, AF647, clone EPR6855; Abcam), or goat anti-rabbit IgG isotype control (#ab150083, AF647, polyclonal; Abcam). All cells were also stained with the Zombie UV fixable viability dye kit (#423107; BioLegend). Cells were acquired on a BD FACSymphony A5 Cell Analyzer (BD Bioscience) and analyzed with FlowJo Software. Briefly, doublet and dead cells were first excluded and then cells were gated as follows: CD20^−^CD3^+^, TCRγδ^−^ TCRαβ^+^, and CD8^−^CD4^+/−^.

### Tfh cell flow cytometry panel on T-blasts

T-blasts were incubated with conjugated mAbs directed against CD3 (#300436, BV570, clone UCHT1; BioLegend), CD8 (#301008, PE, clone RPA-T8; BioLegend), CD154 (#563886, BV421, clone TRAP1; BD Horizon), CD185 (#751293, BUV615, clone RF8B2; BD Horizons OptiBuild), CD278 (#565889, PECF594, clone C398.4A; BD Horizon), and CD279 (#750260, BUV661, clone EH12.1; BD Horizons OptiBuild). All cells were also stained with the Zombie UV fixable viability dye kit (#423107; BioLegend). Cells were acquired on a BD FACSymphony A5 Cell Analyzer (BD Bioscience) and analyzed with FlowJo Software.

### Deep immunophenotyping of lymphocyte cells, in vitro and ex vivo CD3^+^TCRαβ^+^CD8^−^ T cell polarization experiment

Cryopreserved PBMCs and their subpopulations were analyzed with a 28-color flow cytometry panel, as previously described (Payne et al., 2020). Cells were also labeled with antibodies directed against CD45RA (#562886, BV605, clone HI100; BD Horizon), CCR7 (#561143, AF700, clone 150503; BD Pharmingen), CD3 (#562426, BV421, clone UCHT1; BD Horizon), CD8 (#563795, BUV395, clone RPA-T8; BD Horizon), and TCRαβ (#306720, PECy7, clone IP26; BioLegend), TCRγδ (#12-9959-42, PE, clone B1.1; eBioscience), and naive (defined as CD45RA^+^CCR7^+^CD8^−^) and memory (defined as CD45RA^−^CCR7^+/−^CD8^−^) CD3^+^TCRαβ^+^ T cells were isolated (>98% purity) with a FACS Aria cell sorter (BD Biosciences). Isolated naive cells were then cultured with T cell activation and expansion beads (TAE, anti-CD2/CD3/CD28, Miltenyi Biotec) + IL-2 (#IL002, 50 IU/ml; Millipore) to allow proliferation to occur, over a period of 7 days. The cells were then subcultured with TAE beads alone (TH0) or under TH1 (IL-12 [#219-IL-005, 50 ng/ml; R&D Systems]), TH2 (IL-4 [#200-04, 1 IU/ml; Peprotech], TH9 (IL-4 [#200-04, 100 IU/ml; Peprotech], TGF-β1 [#100-21C-10, 2.5 ng/ml; Peprotech]), or TH17 (TGF-β1 [#100-21C-10, 2.5 ng/ml; Peprotech], and IL-1β [#200-01B-10, 50 ng/ml; Peprotech], IL-6 [#200-06-20, 50 ng/ml; PeproTech], IL-21 [#200-21, 50 ng/ml; PeproTech], and IL-23 [#200-23-10, 50 ng/ml; PeproTech]) polarizing conditions. Isolated memory cells were cultured with TAE beads alone (TH0) or under TH1 or TH17 polarizing conditions. After 5 days of polarization, the supernatant was used for assessments of the secretion of IL-2, IL-4, IL-5, IL-6, IL-9, IL-10, IL-13, IL-17A, IL-17F, IFN-γ, and TNF with a cytometric bead array (BD Biosciences). Once the supernatant had been collected, the cells were stimulated with PMA (#P8139-1MG, 100 ng/ml; Sigma-Aldrich)-ionomycin (#I0634-1MG, 750 ng/ml; Sigma-Aldrich) for 6 h, with brefeldin A (#B7651-5MG, 10 mg/ml; Sigma-Aldrich) added after the first 2 h of incubation. For the assessment of intracellular cytokine production, cells were incubated with conjugated mAbs directed against IFN-γ (#564620, BUV737, clone 4S.B3; BD Horizon), TNF (#502924, PerCP, clone Mab11; BioLegend), IL-9 (#560807, PE, clone MH9A3; BD Pharmingen), IL-13 (#563580, BV421, clone JES10-5A2; BD Horizon), IL-4 (#500710, AF488, clone 8D4-8; BioLegend), IL-17A (#512330, BV510, clone BL168; BioLegend), IL-17F (#562264, BV650, clone O33-782; BD Horizon), IL-2 (#566361, BV750, clone MQ1-17H12; BD Horizon), and IL-21 (#50-7219-42, eF660, clone eBio3A3-N2, Thermo Fisher Scientific). All cells were also stained with the Zombie UV fixable viability dye kit (#423107; BioLegend). Cells and beads were acquired on a BD FACSymphony A5 Cell Analyzer (BD Bioscience) and analyzed with FlowJo Software or FCAP Array software. A previously described gating strategy was used (Payne et al., 2020).

### Immunophenotyping of myeloid cells

Cryopreserved PBMCs were incubated with conjugated mAbs directed against HLA-DR (#564040, BUV379, clone G46-6; BD Horizon), CD86 (#612784, BUV737, clone FUN-1; BD Horizon), CD141 (#565321, BV421, clone 1A4; BD Horizon), CD80 (#563315, BV605, clone L307.4; BD Horizon), CD11c (#740966, BV786, clone B-ly6; BD OptiBuild), lin (CD3 [#555332, FITC, clone UCHT1; BD Pharmingen], CD19 [#347543, FITC, clone 4G7; BD Bioscience], CD20 [#347673, FITC, clone L27; BD Bioscience], CD56 [#318304, FITC, clone HCD56; BioLegend]), CD1c (#331514, PerCPCy5.5, clone L161; BioLegend), CD123 (#306006, PE, clone 6H6; BioLegend), CD16 (#302016, PECy7, clone 3G8; BioLegend), CD14 (#557831, APCCy7, clone MøP9; BD Pharmingen), and either CD4 domain 3 (#612963, BUV661, clone SK3; BD Horizon), CD4 domain 4 (#ab196147, AF647, clone EPR6855; Abcam), or goat anti-rabbit IgG isotype control (#ab150083, AF647, polyclonal; Abcam). All cells were also stained with the Zombie UV fixable viability dye kit (#423107; BioLegend). Cells were acquired on a BD FACSymphony A5 Cell Analyzer (BD Bioscience) and analyzed with FlowJo Software. Briefly, doublet and dead cells were first excluded, then subpopulations were defined as follows: total DCs (Lin^−^HLADR^+^CD14^−^CD16^−^), DC subpopulations (mDC1: CD11c^+^CD123^−^CD141^−^CD1C^+^; mDC2: CD11c^+^CD123^−^CD141^+^CD1C^−^; pDC:CD11c^−^CD123^+^), and total Mo (Lin^−^HLADR^+^CD14^+/−^CD16^+/−^).

### Immunophenotyping of CD4^−^CD8^−^ DN T cells

Cryopreserved PBMCs were incubated with conjugated mAbs directed against CD3 (#612940, BUV496, clone UCHT1; BD Horizon), CD4 domain 3 (#612963, BUV661, clone SK3; BD Horizon), CD8 (#612889, BUV805, clone SK1; BD Horizon), TCRαβ (#613014, BUV737, clone T10B9.1A-31; BD Horizon), TCRγδ (#745505, BV711, clone 11F2; BD OptiBuild), CD38 (#303529, BV785, clone HIT2; BioLegend), and CD45RA (#304132, BV570, clone HI100; BD Horizon). All cells were also stained with the Zombie UV fixable viability dye kit (#423107; BioLegend). Cells were acquired on a BD FACSymphony A5 Cell Analyzer (BD Bioscience) and analyzed with FlowJo Software. Briefly, doublet and dead cells were first excluded, then CD3^+^TCRγδ^−^TCRαβ^+^CD8^−^CD4^−^ DN subpopulations were defined as follows: conventional DN (CD38^−^CD45^+/−^), pathogenic DN (CD38^+^CD45^+^), as previously described (Maccari et al., 2021).

### TCR/co-receptor crosslinking in T-blasts

T-blasts were rested for 24 h in pure RPMI (#11875119; Gibco) at 37°C under an atmosphere containing 5% CO_2_. Rested cells were washed twice (cold RPMI) and incubated in the presence or absence of biotinylated anti-CD4 (EPR6855), anti-CD3 (OKT3), or both for 20 min on ice. After two washes (cold RPMI), cells were resuspended at 1 million per ml and plated in a 96-round-bottom-well plate. Cells were incubated with 45 µg/ml of streptavidin (#S0677-1MG; Sigma-Aldrich) or H_2_O_2_ (0.22 mM) at 37°C for either 1, 2, or 5 min. Cells were immediately fixed with 4% formaldehyde (#252549; Sigma-Aldrich). Following permeabilization with saponin, T-blasts were incubated with conjugated mAbs directed against CD3 (#300436, BV570, clone UCHT1; BioLegend), CD8 (#612942, BUV496, clone RPAT8; BD Horizon), and ZAP70 Phospho (Tyr493) (#396005, PE-Cy7, clone A16043E; BioLegend). All cells were also stained with the Zombie UV fixable viability dye kit (#423107; BioLegend). Cells were acquired on a BD FACSymphony A5 Cell Analyzer (BD Bioscience) and analyzed with FlowJo Software.

### Mixed lymphocytes reaction

Allogenic Mo were isolated from healthy donor whole blood using EasySep Direct Human PBMC Isolation Kit (#19654; Stemcell Technologies) and Human Pan Monocyte cell isolation kit (#130-096-537; Miltenyi Biotech) in accordance with the manufacturer’s instructions. Isolated Mo were incubated in the absence or presence of 5 µg/condition of purified antibodies directed against HLA-ABC (clone G46-2.6, #555551; BD Pharmingen) or HLA-DR, DP, and DQ (clone Tü39, #555557; BD Pharmingen) at 37°C under an atmosphere containing 5% CO_2_ for 1 h. T-blasts were incubated with CellTrace Yellow Cell Proliferation Kit (#C34567; Thermo Fisher Scientific) diluted 1:500 in PBS supplemented with 2% FBS at 37°C under an atmosphere containing 5% CO_2_ for 20 min. Five volumes of prewarmed culture media were added and cells were incubated further for 5 min. Cells were centrifuged and resuspended in prewarmed culture media. Mo and T-blasts were counted, and cocultures were plated at a ratio of 1:1. As a control, T-blasts were also incubated in media only or with TAE (anti-CD2/CD3/CD28; Miltenyi Biotec). After 5 days, T-blasts were incubated with conjugated mAbs directed against CD3 (#300436, BV570, clone UCHT1; BioLegend), CD8 (#612942, BUV496, clone RPAT8; BD Horizon), CD20 (#612905, BUV805, clone 2H7; BD Horizon), and TCRαβ (#613014, BUV737, clone T10B9.1A-31; BD Horizon). All cells were also stained with the Zombie UV fixable viability dye kit (#423107; BioLegend). Cells were acquired on a BD FACSymphony A5 Cell Analyzer (BD Bioscience) and analyzed with FlowJo Software.

### Detection of antigen-specific T cells: Activation induced marker assay

We adapted the assay first described by Zaunders and colleagues in 2009 (Zaunders et al., 2009). Isolated cryopreserved PBMCs were cultured in vitro in flat bottom 96-well plates (10^6^ cells/ml) in media only or with tetanus toxoid (gifted from Dr. John Zaunders, Centre for Applied Medical Research, St Vincent's Hospital, Darlinghurst, NSW, Australia, 5 Lfu/ml) or HKCA (#tlrl-hkca, 10^6^ particles/ml; InvivoGen) at 37°C, under an atmosphere containing 5% CO_2_. After 48 h, cells were incubated with conjugated mAbs directed against CD3 (#565491, BV786, clone UCHT1; BD Horizon), CD4 (#612748, BUV737, clone SK3; BD Horizon), CD8 (#563795, BUV395, clone RPA-T8; BD Horizon), CD25 (#347643, FITC, clone 2A3; BD Biosciences), and OX40 (#12-1347-42, PE, clone ACT35; eBioscience). All cells were also stained with the Zombie Aqua fixable viability dye kit (#423102; BioLegend). Cells were acquired on a BD FACSymphony A5 Cell Analyzer (BD Bioscience) and analyzed with FlowJo Software.

### T cell–B cell coculture assay

We adapted the assay previously described (Ma et al., 2005, 2009, 2015). Allogeneic B cells were isolated from blood with EasySep Direct Human B cell Isolation kit (#19674; STEMCELL Technologies) following manufacturer instructions. To prevent proliferation, T-blasts generated from healthy donors and patients were incubated with Mitomycin C (#73274, 150 µM; STEMCELL Technologies) for 1 h in the dark at room temperature. T-blasts (10^4^ cells per well) and allogenic B cells (10^4^ cells per well) were plated at a ratio of 1:1 in a round-bottom 96-well plate. After 7 days, culture supernatants were collected, and IgA, IgG, and IgG secretion was determined using Ig heavy-chain specific ELISAs, as described previously (Avery et al., 2005).

### Phage IP sequencing for microbial antigens (VirScan)

The reactivity of circulating antibodies against common pathogens in plasma samples from the five new patients (P1–P5) and healthy donors (*n* = 20) was profiled with an expanded version of the original VirScan library, as previously described (Béziat et al., 2021). Pooled human plasma for intravenous Ig therapy (Privigen CSL Behring AG) and IgG-depleted human serum (Molecular Innovations, Inc.) were used as additional controls.

### Anti-HPV Luminex serological tests

Plasma samples were sent to the German Cancer Research Center on dry ice for serological analysis. Antibodies against L1 antigens of HPV types 1, 2, 4, 5, 6, 8, 9, 10, 11, 12, 15, 16, 17, 18, 21, 22, 23, 24, 27b, 31, 33, 36, 38, 41, 45, 48, 50, 52, 58, 60, 75, 80, 88, 92, 93, 96, 101, and 103 were analyzed simultaneously with Luminex-based multiplex serological tests, as previously described (Waterboer et al., 2005). In brief, HPV L1 antigens were expressed as recombinant glutathione S-transferase fusion proteins, loaded onto polystyrene beads, and simultaneously presented to primary serum antibodies. The immunocomplexes formed were detected with a biotinylated secondary antibody and streptavidin-R-phycoerythrin as a reporter dye. Sera were tested at a dilution of 1:100, and antigen-specific seropositivity was determined on the basis of predefined cut-off values (Clifford et al., 2007; Michael et al., 2008; Sankaranarayanan et al., 2016).

### scRNAseq of sorted memory CD8^−^ αβ T cells

Memory (defined as CD45RA^−^CCR7^+/−^) CD3^+^CD8^−^TCRαβ^+^ T cells were isolated via FACS and cultured with TAE beads + IL-2 (#IL002, 50 IU/ml; Millipore) for 20 h. Cells were washed three times in cold PBS +2% heat inactivated (HI) FBS and incubated with TotalSeq anti-human hashtag antibodies (A0251-A0256, #394601, #394603, #394605, #394607, #394609, #394611; BioLegend) for 30 min on ice. Hashtagged cells were washed three times in cold PBS +2% HI FBS and passed through a 35-µm nylon mesh cell strainer (#352235; Falcon) to remove aggregates. Cells were resuspended at a concentration of 1,000 cells/µl in cold PBS +2% HI FBS, pooled, and loaded onto a 10x Genomics Chromium B or G chips. Single-cell capture, reverse transcription, and library preparation were performed with the Chromium Single-Cell 3′ Reagent Kits (v3 or v3.1), in accordance with the manufacturer’s instructions. The quality of the cDNA and feature barcode library was assessed with a TapeStation (Agilent). Transcriptome sequencing was performed on the S4 flow cells of an Illumina NovaSeq 6000 sequencer, while hashtag library sequencing was performed on NextSeq 500/550 system with High Output Kit v2.5 (Illumina). Cells from P1/P2/P3 and P4/P5 were processed in two batches of experiments, along with cells from two healthy donors per batch. For comparison, CD3^+^CD4^−^CD8^−^TCRαβ^+^ DN T cells from two healthy donors, one FAS-deficient patient, and one patient with a *STAT3* GOF mutation were isolated and processed similarly.

Hashtag demultiplexing was performed using Seurat with default parameter settings. Hashtag doublets were excluded from subsequent analyses. Cells were further filtered based on standard quality control metrics. Batch correction, unsupervised clustering, pseudobulk PCA, differential expression analysis, and GSEA were performed as described previously (Ogishi et al., 2021). All analyses were conducted in R (version 4.2.2, https://www.R-project.org/). Processed data and relevant codes can be found in Mendeley Data (Guérin et al., 2024b).

### Long-read TCR scRNAseq and prediction of CD4 and CD8 cell type from paired TRA:TRB sequences

Following 10x Genomics Chromium capture and cDNA library preparation (see scRNAseq section), TCR contigs for single cells were obtained by the Repertoire and Gene Expression by Sequencing (RAGE-seq) method (Singh et al., 2019). Briefly, full-length cDNA with 3′ cell barcode and UMI tags from 10x Genomics single-cell gene expression libraries were enriched for TCR cDNA transcripts by capture hybridization and subject to long-read sequencing on the Oxford Nanopore. Long-read sequencing libraries were demultiplexed by cell barcodes obtained from the processing of the paired single-cell gene expression libraries. For each cell barcode, de novo assembly with Canu (version 1.8) (Koren et al., 2017) and error correction plus consensus polishing with Racon (version 1.3.3) (Vaser et al., 2017) were undertaken to obtain a putative set of *TRA*, *TRB*, *TRD*, and/or *TRG* contigs. Contigs were aligned with IgBLAST (version 1.14.0) (Ye et al., 2013) against the human TR IMGT Reference Directory (obtained January 16, 2020) (https://www.imgt.org/vquest/refseqh.html) and corrected for indels relative to the germline genes. Non-productive and truncated contigs were discarded. Finally, clonotypes incorporating the V, J, and CDR3 AA sequences were called for each TR loci for each cell. Where more than one clonotype was identified in a single cell for the same loci, the clonotypes with the highest count were retained.

Basic repertoire features for the single cells were summarized in R (version 4.2.2, https://www.R-project.org/) via RStudio IDE (version 2022.12.0.353, http://www.posit.co/) R packages used for data manipulation and plotting included tidyverse (version 2.0.0), rstatix (version 0.7.2, https://CRAN.R-project.org/package=rstatix), and ggpuborg/package = ggpubr.

To classify single cells as CD4 or CD8 based on paired TRA:TRB paired sequences, an XGBoost model as described in Carter et al. (2019) was used. Modeling was undertaken in R using the xgboost package (version 1.7.3.1, https://CRAN.R-project.org/package=xgboost). Model features included the TRA and TRB CDR3 lengths, TRA and TRB CDR3 charge, CDR3 AA proportions, and one-hot encoding for TRBV and TRAV gene usage. The modeling objective was binary:logistic, and the following parameters were used: nrounds = 1,000; booster = gbtree; eta = 0.01; max_depth = 10; gamma = 1; subsample = 0.8; colsample_bytree = 0.8; and min_child_weight = 5. Training and test sets used the paired single-cell data from Carter et al. (2019) obtained from (https://github.com/JasonACarter/CD4_CD8-Manuscript) after excluding TRA:TRB pairs that were observed across both CD4 and CD8. Training and test datasets were a random subsampling of 14,000 CD4 and 14,000 CD8 across 10 iterations. To set thresholds for the binary logistic regression scores for assigning CD4 and CD8, the labeled test data was used to set the misidentification rate to 5% for each iteration. Cells that fell outside the threshold were considered “non-attributed.” Cell types for the single cells from RAGE-seq were predicted for TRA:TRB pairs for each of the 10 model iterations, and the mean percentage of cells assigned CD4 and CD8 was calculated across the iterations for each donor. Processed data and relevant codes can be found on Mendeley Data (Guérin et al., 2024a).

### Expression analysis of human and murine *CD4* transcripts from public databases

Human bulk RNA-seq datasets were retrieved from the Sequence Read Archive (SRA) using the SRA toolkit (--fastq-dump) for triplicates of double positive CD3^+^ and single positive CD4^+^ thymocytes (PRJNA741323) (Sun et al., 2021), triplicates of CD4^+^ Th1 and Cd4^+^ Treg cells, mDC, pDC, and ncMo (PRJNA418779) (Monaco et al., 2019), as well as non-treated cMo (PRJNA627214) (Panwar et al., 2021). The quality of sequence reads was evaluated using FastQC (Babraham Bioinformatics), and low-quality reads and bases were trimmed using Trimmomatic v.0.33 (Bolger et al., 2014). The reads from biological replicates of each cell type were aligned together to the human hg38 reference assembly using HISAT2 v2.2.1 (Pertea et al., 2016) to obtain higher coverage on the exon splicing junctions. The SAM files were converted to BAM format, sorted, and indexed using Samtools v1.12 (Li et al., 2009). The BAM files were loaded in the Integrated Genome Viewer (Thorvaldsdóttir et al., 2013). The Sashimi plot function was used at the CD4 gene to count the number of spliced reads between exons 1 and 2 (NM_000616) and between exons 1 and 3 or 1 and 4, encompassing alternative transcripts. The proportion of major and alternative CD4 transcripts was calculated relative to the total number of spliced reads from exon 1.

The mouse *Cd4* transcripts expression was investigated using the FANTOM5 CAGE-seq resource (Arner et al., 2015) through the ZENBU portal (Severin et al., 2014). The phase 1 and 2 mouse time course data aligned on the mm9 reference genome assembly were extracted to all tissues and time points for the *Cd4* gene. The CAGE-seq promoter activity mapping identified three transcriptional start sites, with p1 and p2 upstream of exon 1 and corresponding to the main *Cd4* transcript (NM_013488). The third transcriptional start site is located upstream of exon 6 and leads to the expression of a short transcript corresponding to a previously described isoform expressed in mouse brain (Gorman et al., 1987). Relative CAGE expression values are shown for representative cells and tissue.

### Statistics

Statistical analyses were performed using Prism 9 (GraphPad software) and are indicated in the figure legends. Data were analyzed using one-way analysis of variance (ANOVA) with multiple comparisons (Tukey), one-tailed paired, or unpaired Student’s *t* test as indicated in the figure legends.

### Online supplemental material

Fig. S1 shows genetics and in silico analysis as well as the impact of identified *CD4* variants on mRNA and protein expression. Fig. S2 shows the impact of CD4 variants on leukocyte differentiation, function, and TCR signaling. Fig. S3 shows short- and long-read scRNAseq analysis of activated memory CD3^+^TCRαβ^+^CD8^−^ T cells. Fig. S4 shows the effect of CD4 deficiency on NK cells, myeloid cells, Tfh cells, and antiviral antibody responses. Fig. S5 shows in vitro and ex vivo characterization of lymphocyte subsets in CD4-deficient patients. Table S1 shows details of *CD4* variants found in a homozygous state either in patients or in public databases. Table S2 shows T lymphocytes count in patients’ blood. Table S3 shows the immune cell subset count in patients’ blood. Table S4 shows HPV serologies of P2–P5 and P7.

## Supplementary Material

Table S1 shows variants found in homozygous state either in patients or in public databases.

Table S2 shows T lymphocytes count in patients’ blood.

Table S3 shows immune cell subset count in patients’ blood.

Table S4 shows HPV serologies.

SourceData F2 is the source file for Fig. 2.

SourceData F3 is the source file for Fig. 3.

SourceData FS1 is the source file for Fig. S1.

## Data Availability

Minor allele frequencies of *CD4* variants in the general population were retrieved from gnomAD r3.1.1 (https://gnomad.broadinstitute.org/). Public scRNAseq datasets were downloaded from the 10x Genomics website (https://support.10xgenomics.com/single-cell-gene-expression/datasets). For GSEA, gene sets were obtained from MSigDB Collections (http://www.gsea-msigdb.org/gsea/msigdb/index.jsp). Processed data and relevant codes underlying Fig. 4 and Fig. S3 can be found on Mendeley Data for short-read scRNAseq (Guérin et al., 2024b) and long-read TCR scRNAseq (Guérin et al., 2024a), respectively. All other raw and processed data are available upon request from the corresponding authors under a Data Transfer Agreement.
