# Supplementary material for: Helper T cell immunity in humans with inherited CD4 deficiency
Source: J Exp Med. 2024 Apr 1;221(5):e20231044. doi: 10.1084/jem.20231044 (PMC10983808; doi:10.1084/jem.20231044)
Supplement: Table S2 — shows T lymphocytes count in patients’ blood. [file JEM_20231044_TableS2.docx]

| **Laboratory parameter** | **P1** (at 4 y) | **Normal values** | **P2** (at 52 y) | **P3** (at 50 y) | **Normal values** | **P4** | **Normal values** | **P5** | **Normal values** | **P6** (at 23y) | **Normal values** | **P7** (at 45y) | **Normal values** |
| --- | --- | --- | --- | --- | --- | --- | --- | --- | --- | --- | --- | --- | --- |
| **CD3+T (/mm^3^)** | 5,485 | *(900-4,500)* | 922 | 641 | *(515-1,731)* | 9,676 | *(900-4,500)* | N.D. | / | 2,716 | *(650-2,800/μl)* | 1700 | *(743-2,379/μl)* |
| **CD3+CD4+T (/mm^3^)** | 7 | *(900-4,500)* | <1 | 0 | *(286-1,125)* | 0 | *(900-4,500)* | N.D. | / | 0 | *(370-1,336/μl)* | 0 | *(501-1,654/μl)* |
| **CD3+CD8+T (/mm^3^)** | 3,374 | *(300-1,600)* | 571 | 359 | *(118-900)* | 5,612 | *(300-1,600)* | N.D. | / | 2,086 | *(185-1,024/μl)* | 902 | *(133-1,432/μl)* |

**Sup. Table 2:** T lymphocytes count in patients’ blood.

N.D.: not determined
