# Supplementary material for: Helper T cell immunity in humans with inherited CD4 deficiency
Source: J Exp Med. 2024 Apr 1;221(5):e20231044. doi: 10.1084/jem.20231044 (PMC10983808; doi:10.1084/jem.20231044)
Supplement: Table S3 — shows immune cell subset count in patients’ blood. [file JEM_20231044_TableS3.docx]

**Sup. Table 3:** Baseline laboratory tests for inborn errors of immunity in patients' blood

| **Laboratory parameter** | **P1** (at 4 y) | **Normal values** | **P2** (at 52 y) | **P3** (at 50 y) | **Normal values** | **P4** | **Normal values** | **P5** | **Normal values** | **P6** (at 23y) | **Normal values** | **P7** (at 45y) | **Normal values** |
| --- | --- | --- | --- | --- | --- | --- | --- | --- | --- | --- | --- | --- | --- |
| **Leukocytes (/mm^3^)** | N.D. | */* | 6,000 | 11,200 | *(4,000-11,000)* | N.D. | / | N.D. | / | 12.15 | *(4-10.5x10^3^ /μL)* | N.D. | */* |
| **Neutrophils (/mm^3^)** | N.D. | */* | 5,910 | 9,038 | *(1,800-6,900)* | 2,000 | *(1,800-6,900)* | N.D. | / | 6,804,  56 | *(1,600-6,130 /μL, 34-71%)* | 3,872 | *(1,800-6,782/μL)* |
| **Eosinophils (/mm^3^)** | N.D. | */* | 50 | 190 | *(0-630)* | N.D. | / | N.D. | / | 462,  4 | *(40-360 /μL,  0.7-5.8%)* | 213 | *(0-648/μL)* |
| **Basophils (/mm^3^)** | N.D. | */* | 30 | 45 | *(0-110)* | N.D. | / | N.D. | / | N.D. | / | 62 | *(10-64/μL)* |
| **Lymphocytes (/mm^3^)** | 9,219 | *(1,700-6,900)* | 1,210 | 1,053 | *(1,000-4,800)* | N.D. | / | N.D. | / | 3,949 33 | *(1,200-3,740 /μlL 1.2-52%)* | N.D. | */* |
| **Monocytes (/mm^3^)** | N.D. | */* | 560 | 829 | *(180-1,000)* | N.D. | / | N.D. | / | 863 7 | *(240-860 /μL,  4.7-12.5%)* | cMo: 610  ncMo:169 | *(cMo:343-1,104/μL; ncMo: 26-141/μL)* |
| **Hb (g/dL)** | N.D. | */* | 15.9 | 15.8 | *(13.4-16.7)* | N.D. | / | N.D. | / | 14 | *(11.2-15.7 g/dL)* | N.D. | */* |
| **Hemocrite (%)** | N.D. | */* | 44.9 | N.D. | *(39-49)* | N.D. | / | N.D. | / | N.D. | / | N.D. | */* |
| **Platelets (/mm^3^)** | N.D. | */* | 286,000 | 262,000 | *(150,000-400,000)* | N.D. | / | N.D. | / | 333 | *(173-369 x10^3^ /μl)* | N.D. | */* |
| **IgG (mg/dL)** | 1,553 | *(642-2,616 )* | 950 | 667 | *(793-1,590)* | 1,150 | *(793-1,590)* | N.D. | / | 1,000 | *(70-1,600 mg/dL)* | 1.430 | *(70-1,600 mg/dL)* |
| **IgM (mg/dL)** | 254 | *(64-328)* | 480 | 65.6 | *(114-457)* | 105 | *(114-457)* | N.D. | / | 89 | *(40-230 mg/dL)* | 67 | *(40-230 mg/dL)* |
| **IgA (mg/dL)** | N.D. | */* | 48 | 177.6 | *(29-226)* | 75 | *(29-226)* | N.D. | / | 276 | *(70-400 mg/dL)* | 278 | *(70-400 mg/dL)* |
| **IgE (IU/mL)** | 6.08 | *(2-199)* | N.D. | 4 | *(2-199)* | 6 | *(2-199)* | N.D. | / | 1.3 | *(0-90 IU/mL)* | N.D. | */* |
| **CD3+T (/mm^3^)** | 5,485 | *(900-4,500)* | 922 | 641 | *(515-1731)* | 9,676 | *(515-1731)* | N.D. | / | 2,716 | *(650-2,800/μl)* | 1,700 | *(743-2,379/μL)* |
| **CD3+CD4+T (/mm^3^)** | 7 | *(900-4,500)* | <1 | 0 | *(286-1125)* | 0 | *(286-1125)* | N.D. | / | 0 | *(370-1,336/μl)* | 0 | *(501-1,654/μL)* |
| **CD3+CD8+T (/mm^3^)** | 3,374 | *(300-1,600)* | 571 | 359 | *(118-900)* | 5,612 | *(118-900)* | N.D. | / | 2,086 | *(185-1,024/μl)* | 902 | *(133-1,432/μL)* |
| **CD3+CD4-CD8-T (%)** | N.D. | */* | 35 | 33 | *(<2%)* | N.D. | / | N.D. | / | 50.8 % | *(<2%)* | N.D. | */* |
| **CD3+TCRα/β (%)** | N.D. | *./* | 65 | 91.7 | *(90-98%)* | N.D. | / | N.D. | / | 94.4 % | *(90-98%)* | N.D. | */* |
| **CD3+TCRγ/δ (%)** | N.D. | */* | N.D. | 12 | *(2-10%)* | N.D. | / | N.D. | / | 4.96 % | *(2-10%)* | N.D. | */* |
| **CD19+B (/mm^3^)** | N.D. | */* | 208 | 78 | *(169-400)* | 1,935 | *(169-400)* | N.D. | / | 748 | *(80-400/μL)* | 517 | *(48-413/μL)* |
| **CD16+56+ NK (/mm^3^)** | 1,281 | *(100-1,000)* | 156 | 26.56 (%) | *(62-565* *mm3)(10-19%)* | 900 | *(100-1,000)* | N.D. | / | 472 | *(126-841 /μL)* | 603 | *(150-672/μL)* |
| **HLA-DR (%)** | N.D. | */* | 26 | N.D. | */* | N.D. | / | N.D. | / | N.D. | / | N.D. | */* |
| **DHR assay (neutro upon PMA)** | normal | / | normal | normal | */* | N.D. | / | N.D. | / | N.D. | / | N.D. | */* |

N.D.: not determined
