# Supplementary figures and images for: Helper T cell immunity in humans with inherited CD4 deficiency
Source: J Exp Med. 2024 Apr 1;221(5):e20231044. doi: 10.1084/jem.20231044 (PMC10983808; doi:10.1084/jem.20231044)

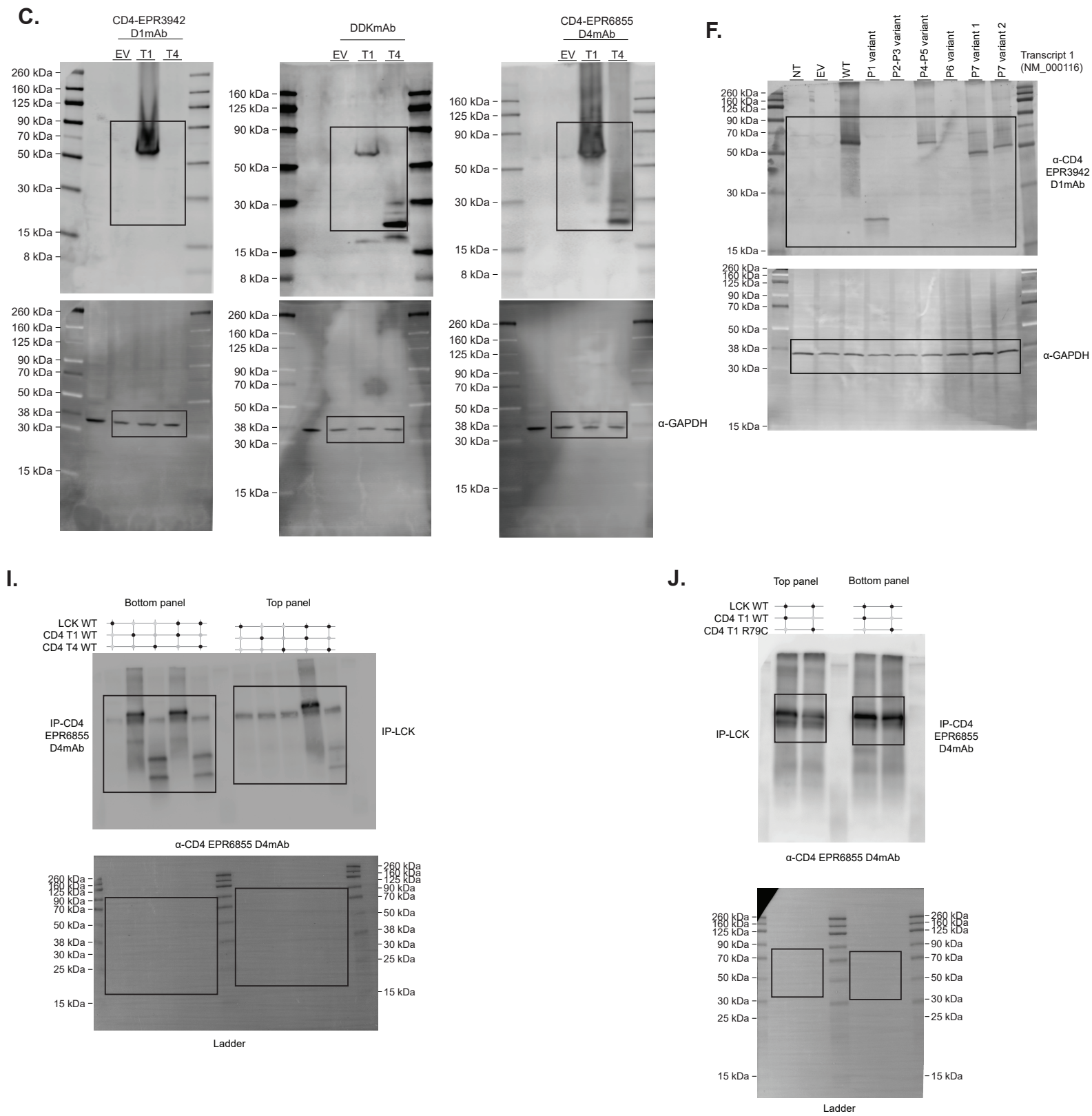

**Fig. 2**

Supplement: SourceData F2 — is the source file for Fig. 2. [file JEM_20231044_SourceDataF2.pdf]

C.

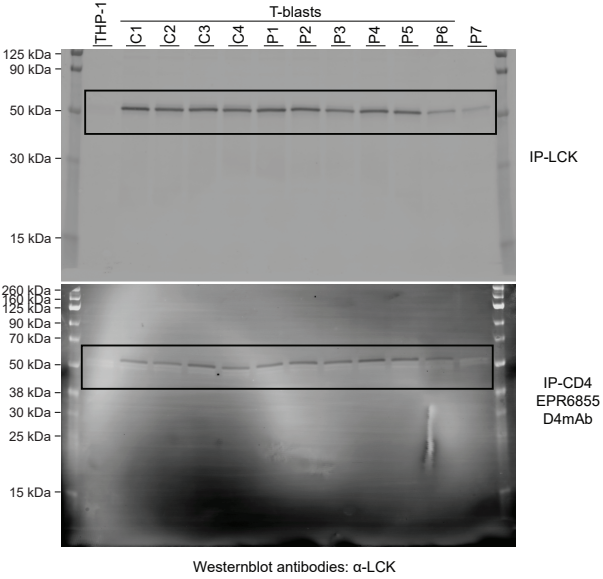

Fig. 3

Supplement: SourceData F3 — is the source file for Fig. 3. [file JEM_20231044_SourceDataF3.pdf]

J.

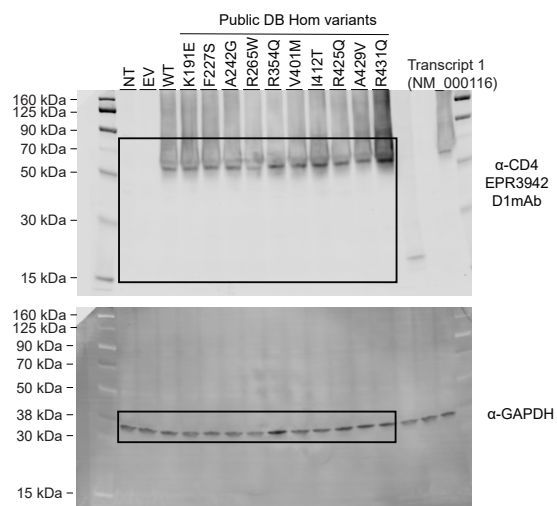

Sup. Fig. 1

Supplement: SourceData FS1 — is the source file for Fig. S1. [file JEM_20231044_SourceDataFS1.pdf]
